# Immune function differs among tropical environments but is not downregulated during reproduction in three year-round breeding equatorial lark populations

**DOI:** 10.1007/s00442-021-05052-0

**Published:** 2021-10-12

**Authors:** Henry K. Ndithia, Kevin D. Matson, Muchane Muchai, B. Irene Tieleman

**Affiliations:** 1 Ornithology Section, Department of Zoology, National Museums of Kenya, P.O. Box 40658, Nairobi, 00100 GPO Kenya; 2 Groningen Institute for Evolutionary Life Sciences, University of Groningen, P.O. Box 11103, 9700 CC Groningen, The Netherlands; 3 Wildlife Ecology and Conservation, Environmental Sciences Group, Wageningen University, Droevendaalsesteeg 3a, 6708 PB Wageningen, The Netherlands; 4 Department of Clinical Studies (Wildlife and Conservation), College of Agriculture and Veterinary Sciences, University of Nairobi, Box 30197-00100, Nairobi, Kenya

**Keywords:** Chick-feeding, Non-breeding, Immune function, Environmental conditions, Tropics

## Abstract

Seasonal variation in immune function can be attributed to life history trade-offs, and to variation in environmental conditions. However, because phenological stages and environmental conditions co-vary in temperate and arctic zones, their separate contributions have not been determined. We compared immune function and body mass of incubating (female only), chick-feeding (female and male), and non-breeding (female and male) red-capped larks *Calandrella cinerea* breeding year-round in three tropical equatorial (Kenya) environments with distinct climates. We measured four immune indices: haptoglobin, nitric oxide, agglutination, and lysis. To confirm that variation in immune function between breeding (i.e., incubating or chick-feeding) and non-breeding was not confounded by environmental conditions, we tested if rainfall, average minimum temperature (*T*_min_), and average maximum temperature (*T*_max_) differed during sampling times among the three breeding statuses per location. *T*_min_ and *T*_max_ differed between chick-feeding and non-breeding, suggesting that birds utilized environmental conditions differently in different locations for reproduction. Immune indices did not differ between incubating, chick-feeding and non-breeding birds in all three locations. There were two exceptions: nitric oxide was higher during incubation in cool and wet South Kinangop, and it was higher during chick-feeding in the cool and dry North Kinangop compared to non-breeding birds in these locations. For nitric oxide, agglutination, and lysis, we found among-location differences within breeding stage. In equatorial tropical birds, variation in immune function seems to be better explained by among-location climate-induced environmental conditions than by breeding status. Our findings raise questions about how within-location environmental variation relates to and affects immune function.

**Supplementary Information:**

The online version contains supplementary material available at 10.1007/s00442-021-05052-0.

## Introduction

Seasonal variation in immune function can be attributed to life history trade-offs and to variation in environmental conditions (Martin et al. 2008; Tieleman 2018). But these latter two phenomena are known to strongly co-vary in temperate and arctic areas, where seasonal variation in immune function has been studied. Thus, disentangling the effects of life history trade-offs and environmental variation on immune function has presented a challenge to ecologists and requires studies in systems where these factors do not co-vary.

Certain events associated with an organism’s life history, such as reproduction and migration, can be resource demanding (Piersma 1997; Martin et al. 2008). Consequently, these events may result in trade-offs with the immune system, a critical component of self-maintenance and survival (Ilmonen et al. 2000; Buehler et al. 2008; Martin et al. 2008; Hegemann et al. 2012a, b; Horrocks et al. 2012a). Seasonal variation in constitutive innate immune function in birds from temperate and arctic zones has been attributed to such trade-offs (Martin et al. 2008; Hegemann et al. 2012a). Yet other studies provide contrary evidence showing that immune function is maintained in the face of reproduction (Tieleman et al. 2019; Schultz et al. 2020) and other supposedly competing physiological processes, e.g., endocrinological changes (e.g. Vindevogel et al. 1985; Allander and Sundberg 1997; Christe et al. 2000; Møller et al. 2003; Alonso-Alvarez et al. 2007).

Immune function also varies with the abiotic conditions of an animal’s environment (Lowen et al. 2007; Rubenstein et al. 2008; Sehgal et al. 2011; Horrocks et al. 2012b, 2015; Zamora-Vilchis et al. 2012; Schultz et al. 2020), and a single species can mount different immune responses depending on geographical location (Ardia 2007). This type of immunological variation may reflect resource availability, “pathogen pressure”, or some combination of the two (Schultz et al. 2020; van Veelen et al. 2020). Pathogen pressure encompasses the abundance and diversity of parasites, pathogens, and even commensal microorganisms in the environment (Sheldon and Verhulst 1996; Christe et al. 2001; Møller et al. 2003; Tschirren and Richner 2006; Horrocks et al. 2011, 2012b) and on the animal itself (Horrocks et al. 2012a). Warm temperatures are known to provide a conducive environment for growth, development, and reproduction of many microorganisms and parasites (Sehgal et al. 2011; Zamora-Vilchis et al. 2012), and rain can correlate positively with microbial load (Atherholt et al. 1998; Landesman et al. 2011; Tieleman et al. 2019). Parasites and microbial communities may vary in space (Bensch and Åkesson 2003; Angel et al. 2010; Froeschke et al. 2010; Knowles et al. 2010). Our three study locations with different climates occur along an aridity gradient where the antigen exposure hypothesis, which predicts reduced microbial abundance in arid environments (Horrocks et al. 2012b), may apply.

Many birds living at or near the equator exhibit life history traits, such as year-round breeding, that are ideally suited for disentangling the effects of life history trade-offs and environmental variation on immune function. Year-round breeding means reproduction is not tightly confounded with predictable intra-annual variation, as it is at mid-to-higher latitudes. In addition, some tropical equatorial regions are characterized by large variations in environmental conditions over short distances (Ndithia et al. 2017a); such variation can be exploited for confirming whether environmental conditions affect immune function. Tropical equatorial bird species that are distributed widely and that breed year-round allow for simultaneous comparisons of variation in immune function in breeding and non-breeding individuals within and among environments. However, seasonal or temporal variation in immune function in tropical equatorial birds is poorly studied in comparison to their temperate and arctic zone counterparts.

To understand the roles of reproduction and the potential role of environmental conditions in influencing immune function, we studied three populations of year-round breeding red-capped larks *Calandrella cinerea* living in three locations in equatorial Kenya: South Kinangop (cool and wet), North Kinangop (cool and dry) and Kedong (warm and dry). These three locations are geographically close, but climatically distinct (Ndithia et al. 2017a). They have distinct differences in average annual rainfall, average minimum temperature (*T*_min_), and average maximum temperature (*T*_max_), and they are characterized by large intra- and inter-annual variations in the quantity and timing of rainfall (Ndithia et al. 2017a). By residing and breeding in the three locations, our study species offers the opportunity to explore immunological variation (1) among three breeding statuses within each location and (2) among the three climatically distinct locations. Although our study species and system also allow for exploring how immune defenses vary with changing environmental conditions within each location, the current study is not designed to address this question.

We investigated if immune function and body mass differed among red-capped larks in three different breeding statuses and from three climatically distinct environments that are generally permissive of year-round breeding (Ndithia et al. 2017a). The breeding status included incubation (females only), chick-feeding (females and males), and non-breeding (females and males). Based on our findings in Ndithia et al. (2017a), we expected that environmental conditions (rain, *T*_min_, *T*_max_) would not differ between the time points at which we sampled breeding (i.e., incubating and chick-feeding) birds and the time points at which we sampled non-breeding ones. Hence, we expected to be able to exclude environmental conditions as confounding factors in explaining any reproduction-associated variation in immune function. Based on resource trade-offs, we expected non-breeding birds within each location to generally have more robust immune function (Nelson and Demas 1996; Bentley et al. 1998; Martin et al. 2008) and higher body mass (Moreno 1989) compared to breeding ones. Based on the antigen exposure hypothesis, which predicts reduced microbial abundance in arid environments (Horrocks et al. 2012b), we expected immune function to decrease along a gradient of aridity from the cool and wet South Kinangop to the cool and dry North Kinangop and the warm and dry Kedong. Because warm temperatures are known to promote growth of pathogens (Sehgal et al. 2011; Zamora-Vilchis et al. 2012), we expected aridity to override temperature effects.

## Methods

### Study species

The red**-**capped lark is a small (mean mass (g): 25.6 ± 1.54 (SD), *n* = 66) gregarious bird in the family *Alaudidae* that occurs in grasslands with short grass and patches of bare ground. Its distribution ranges from lowland savanna ca. 1200 m above sea level (ASL) to highland grasslands ca. 2600 m ASL (Zimmermann et al. 1999). The species feeds on invertebrates including beetles, wasps, caterpillars, butterflies and moths, earthworms, grasshoppers, and occasionally on grass seeds (H.K.N pers. obs.). Food supply fluctuates throughout the year in all locations, but food does not appear to be limiting at any given time. The warm and dry Kedong has the highest food abundance; the cool and dry North Kinangop has the lowest (Ndithia et al. 2017a). Red-capped larks breed year-round, building an open**-**cup nest on the ground, often next to a scrub or grass tuft. Females lay two eggs (mean: 2.0 ± 0.0 (SD), *n* = 59), which are incubated for 10–12 days by females only. Both parents feed the nestlings for about 10 days (H.K.N. pers. obs.). The species can breed in all calendar months, but in every year there are months in which no breeding occurs (Ndithia et al 2017a). Color ring re**-**sightings suggest that birds reside year**-**round within locations.

### Study areas and environmental conditions

From January 2011 to March 2014, we worked year-round and simultaneously in three geographically close, but climatically distinct locations: cool and wet South Kinangop, cool and dry North Kinangop and warm and dry Kedong (Electronic Supplementary Material, ESM Table 1a, ESM Table 1b, Ndithia et al. 2017a).

### Field sampling and recording of environmental abiotic variables

In each location, we used mist nets throughout the year to catch adults of both sexes that were not breeding, as determined by their flocking behavior (breeding birds are paired and territorial; there is no particular non-breeding period). We used cage traps to catch females during egg incubation and both sexes during chick-feeding, also year-round (again, there was no particular breeding period).

Because only females in this species incubate eggs, we separately analysed using similar approaches two datasets, sexes combined and females only, to test for effects of reproduction (sexes-combined dataset: *n*_South Kinangop_ = 32, *n*_North Kinangop_ = 37, *n*_Kedong_ = 79; females-only dataset: *n*_South Kinangop_ = 26, *n*_North Kinangop_ = 28 and n_Kedong_ = 62), and of environmental conditions (sexes-combined dataset: *n*_South Kinangop_ = 32, *n*_North Kinangop_ = 37 and *n*_Kedong_ = 82; females-only dataset: *n*_South Kinangop_ = 28, *n*_North Kinangop_ = 32 and *n*_Kedong_ = 65) on immune function. We avoided lumping incubation and chick-feeding into one breeding category when comparing the effect of breeding on immune function because if there are differences between incubation and chick-feeding in females (as found in Dehnhard and Hennicke 2013), this would negatively affect the analyses of effects of sex and of breeding/non-breeding. For instance, it would be inappropriate to compare males (for which breeding only involves chick-feeding) with females (for which breeding involves incubation and chick-feeding) (ESM Table 2 contains details on sample sizes per breeding status, location, and sex).

Our nest searching strategy included observing breeding behavior (e.g., transport of nest materials or food, breeding-related alarm calls, and nervous parental behavior) and routinely walking plots to flush parents incubating eggs or brooding young (Ndithia et al. 2017a). We spent an average of 134 person-hours per month searching for nests in the three locations combined; Ndithia et al. (2017a) provides details on searching effort per location. The search efforts were required for collecting samples for immunological analyses and for monitoring reproduction (these two activities occurred simultaneously). In our two datasets (sexes-combined and females-only) of individually-marked birds sampled during specific breeding statuses (non-breeding, incubation and chick-feeding) throughout the year for 38 months, each sampled animal was a unique individual (no repeated measures of individuals). Because breeding occurred year-round, and we sampled the three study locations at the same time, we did not expect the plasma storage time (time between plasma collection in the field and analyses in the laboratory) to co-vary with variables (i.e., location, breeding status and sex). Plasma storage time only co-varied with breeding status for both datasets (sexes-combined, and females-only) for haptoglobin, and with location and breeding status for both datasets for nitric oxide. There was no co-variation with plasma storage time for these variables for the rest of the immune indices (*F* < 3.17, *P* > 0.07).

From each individual, we collected a blood sample for immunological analyses from a needle puncture of the brachial vein using heparinized capillary tubes. We transferred these samples to microcentrifuge tubes, temporarily stored them on ice, and centrifuged them at the end of each fieldwork day. We stored the plasma fraction in the freezer at − 20 °C for future analyses. We used a weather station (Alecto WS**-**3500, Den Bosch, Netherlands) in each location to obtain monthly total rainfall (mm), *T*_min_ (^°^C)_,_ and *T*_max_ (^°^C). We worked in one plot in the cool and wet South Kinangop that was 5.2 km from its corresponding weather station, in three plots in the warm and dry North Kinangop that averaged 2.7 km from their weather station, and in four plots in the warm and dry Kedong that averaged 8.5 km from their weather station.

### Immune assays

Haptoglobin is an acute phase protein that scavenges haemoglobin. Haemoglobin can be released into circulation by haemolysis or normal red blood cell turnover (Quaye 2008); outside of erythrocytes haemoglobin is toxic (Alayash 2004). Circulating concentrations of haptoglobin (mg ml^−1^) increase in response to inflammation, infection, or injury (Quaye 2008). We determined haptoglobin concentration using an assay that measures the haem**-**binding capacity of plasma (TP801; Tridelta Development limited, Maynooth, Ireland). We followed the manufacturer’s instructions and incubated the assay reaction at 30 °C for 5 min. More details are available from Matson et al. (2012). Each of the three assay plates included a standard that we ran in duplicate (Matson et al. 2012). Mean within**-**plate coefficient of variation (CV) equalled 2.4%; among**-**plate CV equalled 2.7%.

Nitric oxide is a multifunctional signalling molecule that, among other roles, is important for modulating inflammatory processes and destroying parasites, virus**-**infected cells, and tumour cells. Therefore, the molecule provides information about the physiological condition of animals (Sild and Hõrak 2009). We determined nitric oxide (mmol ml^−1^) production through the reduction of nitrate to nitrite by copper**-**coated cadmium granules, followed by color development with Griess reagent (Promega; Sild and Hõrak 2009) and absorbance measurement at 542 nm (Versamax, Molecular Devices Sunnyvale, California, US).

Natural antibodies (hemagglutination) and complement (hemolysis) are constitutively present as part of the innate immune system, which provides a first line of defence (no previous exposure to particular antigens required) against infectious agents (Matson et al. 2005). Natural antibodies can bind to and agglutinate a range of antigens and can also initiate the complement enzyme cascade that leads to cell lysis (Congdon et al. 1969; Greenberg 1985; Reid et al. 1997; Carroll and Prodeus 1998; Ochsenbein et al. 1999; Belperron and Bockenstedt 2001; Matson et al. 2005). We quantified lysis (presence/absence) and agglutination (titres) against rabbit red blood cells (Envigo, Belton, UK) following the protocol of Matson et al. (2005). Blind to sample and plate identity, we scored lysis and agglutination titres at least twice, and assigned half scores to samples that showed intermediate results. For agglutination, we used the mean value in statistical analyses if the first two scores was one or less than one titre apart, and we scored a third time and used the median value if scores were more than one titre apart (Matson et al. 2005). Since there were many zero values, we treated lysis as either present or absent in a sample. We ran two samples from a standard plasma source in all plates. For lysis, mean among**-**plate CV equalled 18.6% and mean within**-**plate CV equalled 9.8%. For agglutination, among**-**plate CV equalled 9.7% and mean within**-**plate CV equalled 7.7%.

### Statistical analyses

To test if immune function and body mass are determined by breeding status and/or environmental conditions in larks of both sexes (i.e., sexes-combined dataset) from the three locations, we constructed generalized linear models (glm) with each immune index or mass as a dependent variable and with breeding status, location, sex, and the two-way interactions as explanatory variables (ESM Table 3a (i)). We included plasma sample redness for haptoglobin and plasma storage time for all models (haptoglobin, nitric oxide, agglutination and lysis) from the starting to the final models to control for these covariates. We log-transformed haptoglobin and nitric oxide values to obtain normality. We used a normal (Gaussian) distribution for analyses of log haptoglobin, log nitric oxide, agglutination and body mass, and we used a binomial distribution for the analysis of lysis (presence/absence) due to many zero values in the data.

The haptoglobin assay can be affected by hemolysis (Matson et al. 2012). Therefore, for the sexes-combined dataset, we pre-scanned samples at 450 nm to enable us to statistically correct for plasma sample redness. Using regression, we found that log haptoglobin was affected by plasma sample redness at 450 nm (*F*_1, 127_ = 8.49, *P* = 0.004). Since plasma samples ranged in storage time (81–1275 days), we also tested for the effect of this time on log haptoglobin (*F*_1, 127_ = 12.54, *P* = 0.001), log nitric oxide (*F*_1, 130_ = 1.10, *P* = 0.30), agglutination (*F*_1, 139_ = 10.76, *P* = 0.001) and lysis (*X*^2^ = 38.96, d.f. 1, *P* < 0.001).

To check whether or not environmental conditions differed between the time points of sampling breeding and non-breeding birds (i.e., sexes-combined dataset), in which case they could confound the possible effects of breeding on immunity, we tested if total rain (mm), *T*_min_ (°C), and *T*_max_ (°C) differed between the sampling times of non-breeding and chick-feeding in female and male birds in the three locations. We constructed models with each of these environmental conditions as dependent variables and with sampling time points (breeding vs non-breeding), location, sex, and the two-way interactions as explanatory variables (ESM Table 3b (i)). Because we had only a monthly average for each environmental condition per month but had sampled multiple bird individuals for immune function per month, we matched the time point (month) of an immune measurement of an individual bird with the corresponding total monthly rainfall, *T*_min_, and *T*_max_.

To test for differences in immune function and body mass among non-breeding, incubating, and chick-feeding females in the three locations (i.e., females-only dataset), we built separate models for each immune index and for body mass (dependent variables) with breeding status (non-breeding, incubating and chick-feeding), location, and the interaction as explanatory variables (ESM Table 3a (ii)). Again, we also added plasma sample redness for haptoglobin and plasma storage time for all models (haptoglobin, nitric oxide, agglutination and lysis) to control for these covariates from the starting to the final models. We log-transformed haptoglobin and nitric oxide data to obtain normality. We used a normal (Gaussian) distribution for analyses of log haptoglobin, log nitric oxide, agglutination and body mass, and a binomial distribution for the analyses of lysis (presence/absence). We found no significant effects of plasma storage time (*F*_1, 100_ = 3.41, *P* = 0.07) or plasma sample redness at 450 nm (*F*_1, 100_ = 0.49, *P* = 0.49) on log haptoglobin. Plasma storage time did not affect log nitric oxide (*F*_1, 105_ = 0.99, *P* = 0.32) but did affect agglutination (*F*_1, 98_ = 6.48, *P* = 0.01) and lysis (*X*^2^ = 21.48, d.f. = 1, *P* < 0.001).

With the female only dataset, we also checked whether environmental conditions differed among the time points at which we sampled the three breeding statuses (non-breeding, incubation and chick-feeding). We tested if total monthly rain, *T*_min_, and *T*_max_ differed among sampling time points at which we measured non-breeding, incubating, and chick-feeding females in the three locations. Like in the dataset with both sexes, we matched the month in which we sampled each immune index for an individual bird with the corresponding total monthly rainfall, *T*_min_, and *T*_max_. We built models with each of the different environmental conditions as the dependent variable and with the three groups of sampling time points of breeding status, location and their interaction as explanatory variables (ESM Table 3b (ii)).

We simplified models using backward elimination (*P* > 0.05 as selection criterion), iteratively deleting the least significant term from a model until we arrived at a final model. However, we always retained the main effects of interest (breeding status, location and sex (where applicable) and the methodological covariates (plasma sample redness (where applicable) and plasma storage time) in the models, i.e., only the interactions were eliminated when *P* > 0.05. We used type III sum of squares in the ANOVA summary of results to test main effects in the presence of interactions (Mangiafico 2015). For all analyses, we tested and confirmed that the residuals of the final models observed the assumptions of normality and homoscedasticity of variance through graphical and statistical methods. Whenever an interaction was significant, we made a new variable consisting of all variable combinations from that interaction, conducted a Tukey’s post hoc test on this new variable, and reported significant post hoc test results. We used R statistical software (version 3.0.3; R Development Core Team 2014) for all analyses.

## Results

### Immune function and body mass in non-breeding and chick-feeding red-capped larks from three locations: sexes-combined analyses

**Fig. 1:**
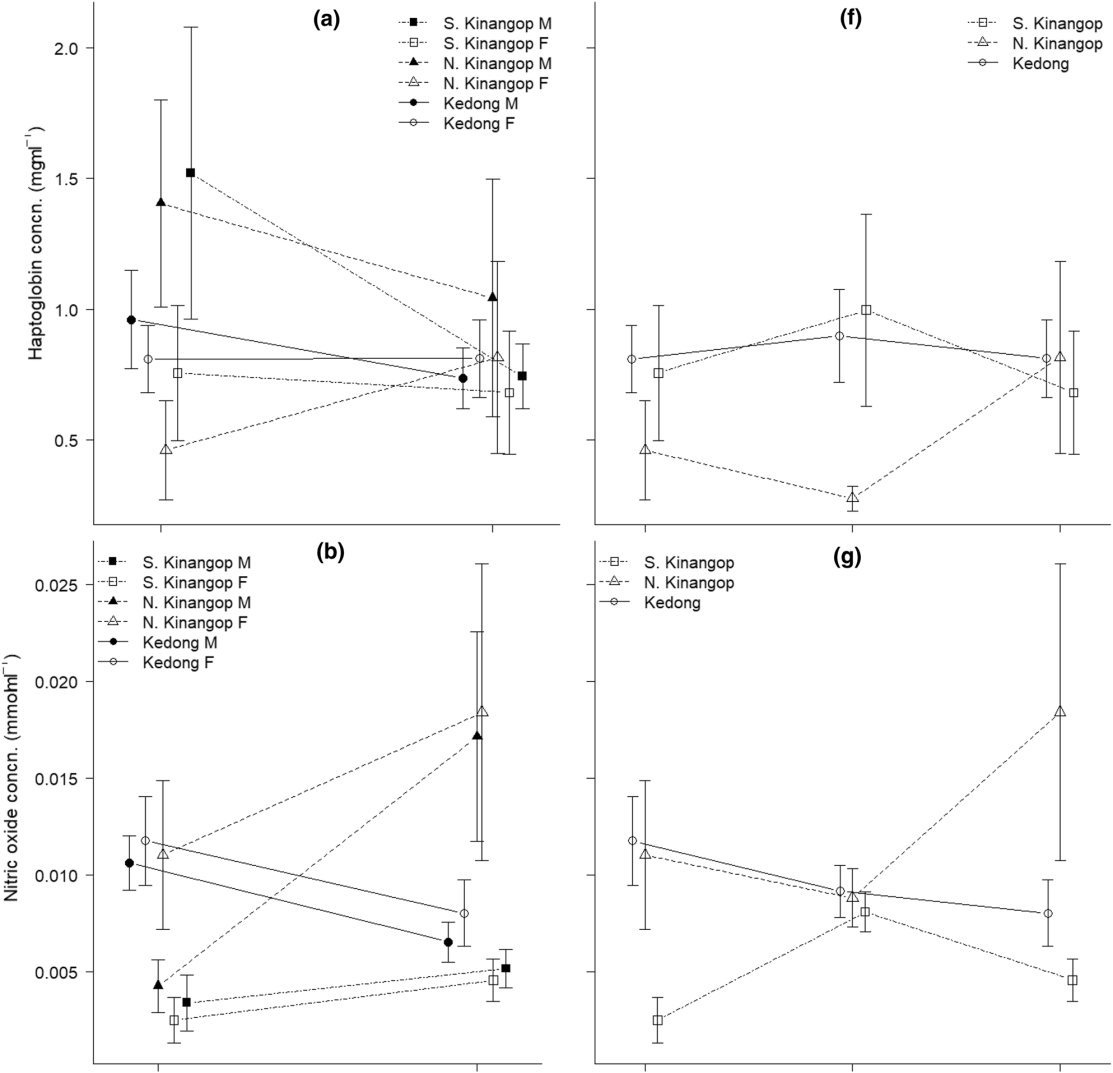

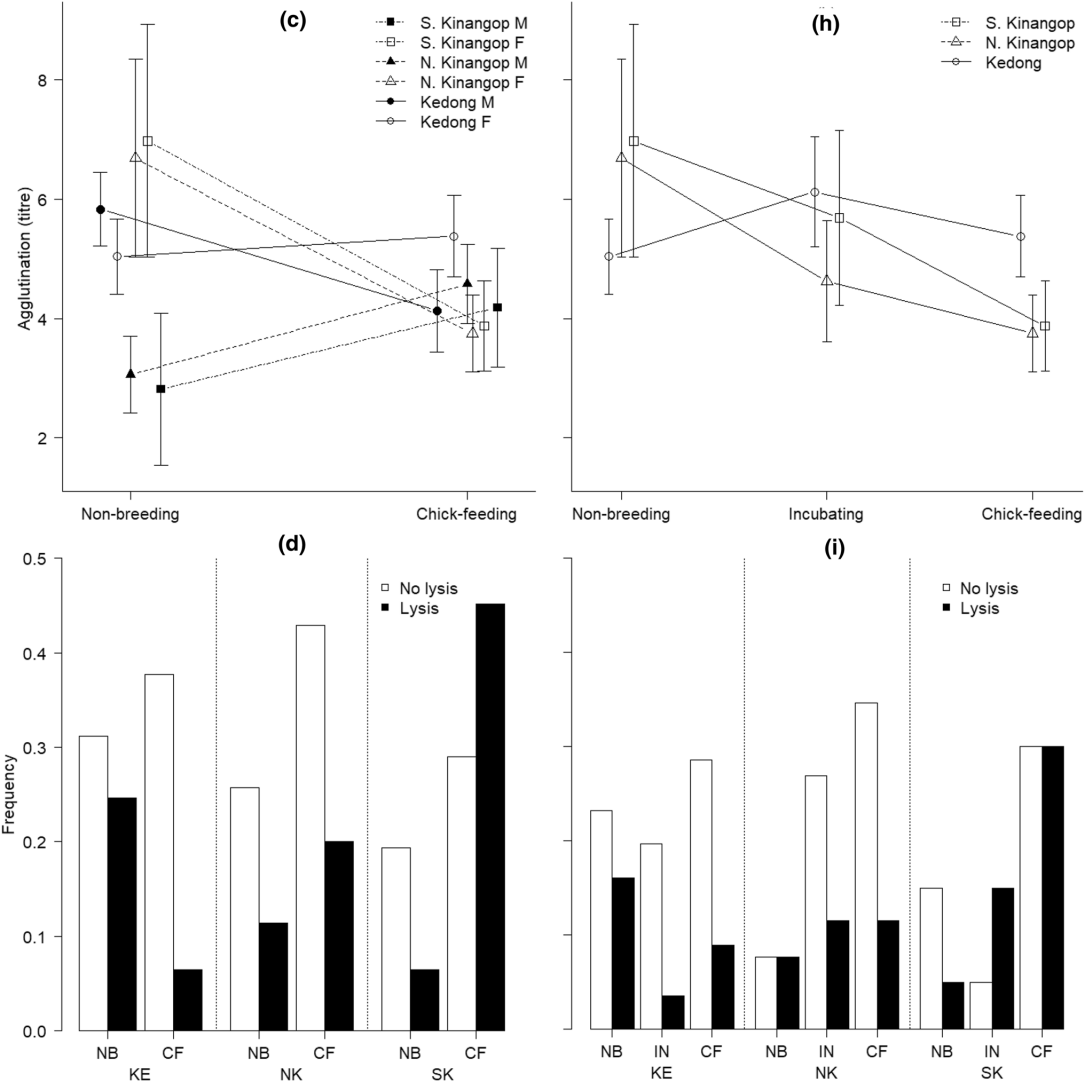

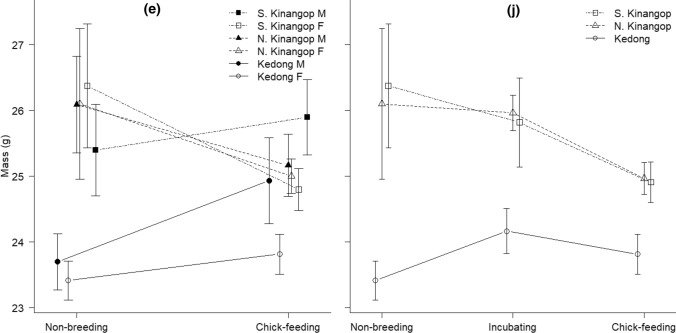
**a** Haptoglobin (mean ± SE, mg ml^−1^), **b** nitric oxide (mean ± SE, mmol ml^−1^), **c** agglutination (mean ± SE, titre), **d** frequency of lysis (presence/absence), **e** mass (g) in non-breeding and chick-feeding males and females, and **f** haptoglobin (mean ± SE, mg ml^−1^), **g** nitric oxide (mean ± SE, mmol ml^−1^), **h** agglutination (mean ± SE, titre), **i** frequency of lysis (presence/absence), **j** mass (g) in in non-breeding, incubating and chick-feeding females of our study species, red**-**capped larks *Calandrella cinerea*, in South Kinangop (cool and wet), North Kinangop (cool and dry) and Kedong (warm and dry) in equatorial Kenya that we studied from January 2011 to March 2014. For the graphs of frequency of lysis (**d** and **i**), the axis include NB = non-breeding, IN = incubating and chick-feeding, and the axis title include study locations, KE = Kedong, NK = North Kinangop and SK = South Kinangop

We found no consistent differences between non-breeding and chick-feeding individuals for haptoglobin, nitric oxide, agglutination, or lysis (Fig. 1a–d). Breeding status did not significantly affect haptoglobin and agglutination in males and females in any of the locations, but we found a significant interaction of breeding status × location for nitric oxide and lysis (Table 1). Although there was a significant interaction of location × sex for haptoglobin (Fig. 1a, Table 1), post hoc tests revealed no significant differences among groups (all *t* < 2.61, all *P* > 0.07). Further exploration of the significant interaction of breeding status × location for nitric oxide revealed that values were higher during chick-feeding than during non-breeding in the cool and dry North Kinangop (*t* = 3.39, *P* = 0.007). Among locations during non-breeding, birds in the warm and dry Kedong had higher nitric oxide than those in the cool and wet South Kinangop (*t* = 4.70, *P* < 0.001) and the cool and dry North Kinangop (*t* = 2.86, *P* = 0.04). Among locations during chick-feeding, birds in the cool and dry North Kinangop had higher nitric oxide than those in the cool and wet South Kinangop (*t* = 4.13, *P* < 0.001) and the warm and dry Kedong (*t* = 2.80, *P* = 0.04). All other pairwise comparisons for nitric oxide were non-significant (all *t* < 2.12, all *P* > 0.22). Sex did not affect nitric oxide (Table 1). Although location was marginally significant for agglutination, post hoc test did not reveal any significant differences among locations (all *t* < 2.33, all *P* > 0.05). The significant interaction of breeding status × location for lysis (Fig. 1d, Table 1) also did not reveal any significant differences among breeding status and location (all *z* < 2.66, all *P* > 0.06).

**Table 1:** Results of models examining variation in immune function between chick-feeding and non-breeding male and female (sexes-combined dataset) red**-**capped larks *Calandrella cinerea* in the cool and wet South Kinangop, the cool and dry North Kinangop and the warm and dry Kedong in equatorial Kenya

Immune index	Explanatory variable	DF	*F*	*P*
Haptoglobin (mg ml^−1^)	Breeding status × location	2, 117	0.67	0.51
	Breeding status × sex	1, 119	1.75	0.19
	*Location *×* sex*	*2, 120*	*3.14*	***0.047***
	*Breeding status*	*1, 120*	*1.19*	*0.28*
Nitric oxide (mmol ml^−1^)	Breeding status × sex	1, 121	0.29	0.59
	Location × sex	2, 122	0.93	0.40
	*Breeding status *×* location*	*2, 125*	*9.23*	** < *0.001***
	*Sex*	*1, 124*	*0.05*	*0.83*
Agglutination (titre)	Breeding status × location	2, 130	0.18	0.84
	Location × sex	2, 132	0.80	0.45
	Breeding status × sex	1, 134	0.56	0.45
	*Breeding status*	*1, 135*	*0.14*	*0.71*
	*Location*	*2, 135*	*3.08*	***0.048***
	*Sex*	*1, 135*	*0.69*	*0.41*
Lysis (presence/absence)	Breeding status × sex	1, 132	0.15	0.70
	Location × sex	2, 133	1.45	0.48
	*Breeding status *×* location*	*2, 135*	*12.31*	***0.002***
	*Sex*	*1, 135*	*0.05*	*0.82*
Body mass (g)	Location × sex	2, 134	0.52	0.59
	Breeding status × sex	1, 136	1.83	0.18
	*Breeding status *×* location*	*2, 137*	*3.24*	***0.04***
	*Sex*	*1, 137*	*2.64*	*0.11*

These are *F *statistics (or *X*^2^ statistic in case of lysis) and *P* values for parameters in the model at the last stage before parameter elimination, or in the final model, and in their order of removal. Methodological covariates and constituent main effects of remaining interactions were not removed, and are not shown. Terms that are in the final model are in italics. Data of haptoglobin and nitric oxide were log transformed to obtain normality. *P* values < 0.05 are indicated in bold

The interaction of breeding status × location for body mass was significant, but there was no significant effect of sex on mass (Fig. 1e, Table 1). Post hoc tests for this significant interaction revealed that during non-breeding, larks weighed less in warm and dry Kedong than in the cool and wet South Kinangop (*t* = 3.60, *P* = 0.004) and in the cool and dry North Kinangop (*t* = 4.90, *P* < 0.001). All other pair-wise comparisons were non-significant (all *t* < 2.10, all *P* > 0.23).

### Rainfall and temperature at the time of sampling non-breeding and chick-feeding red-capped larks in three locations: sexes-combined analyses

**Fig. 2:**
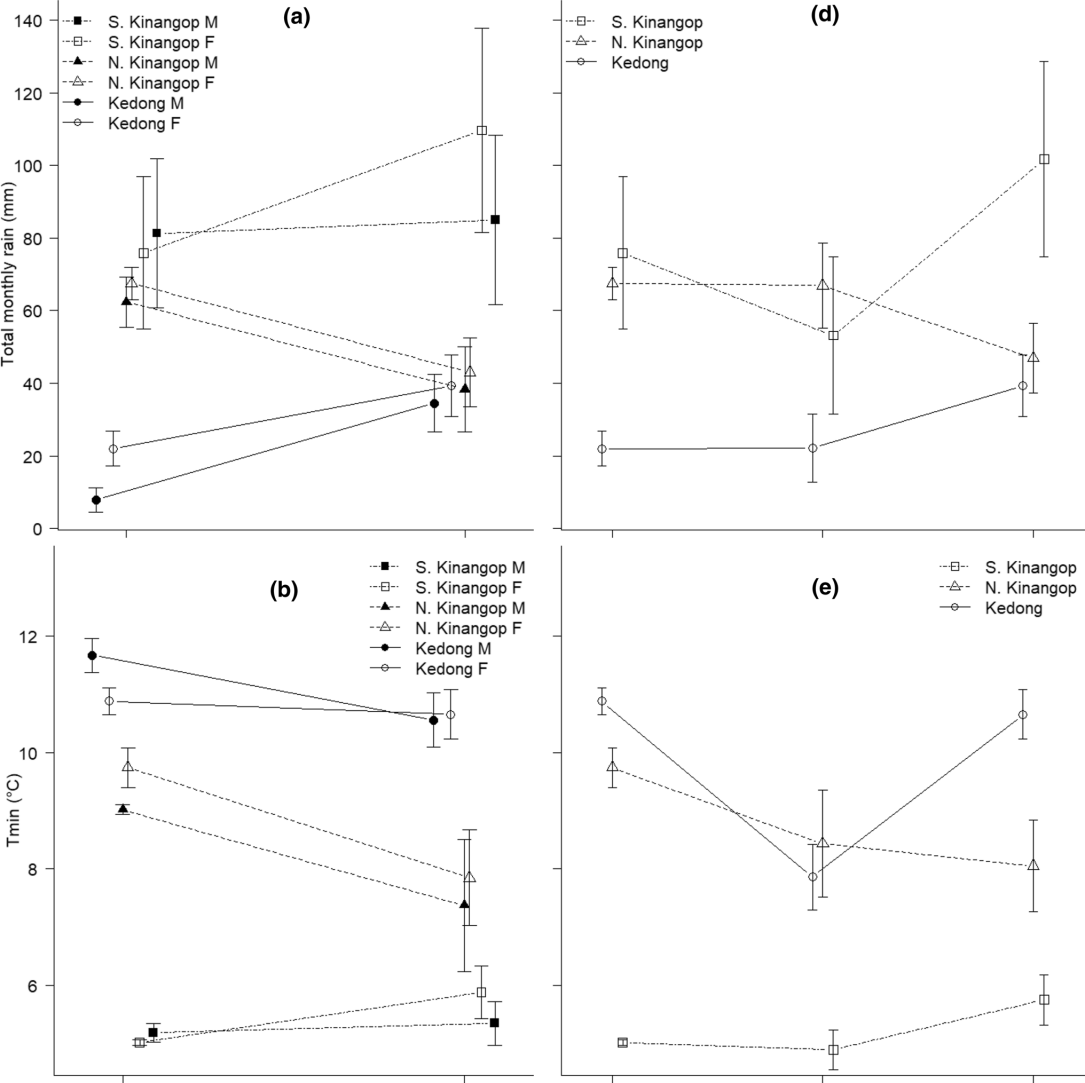

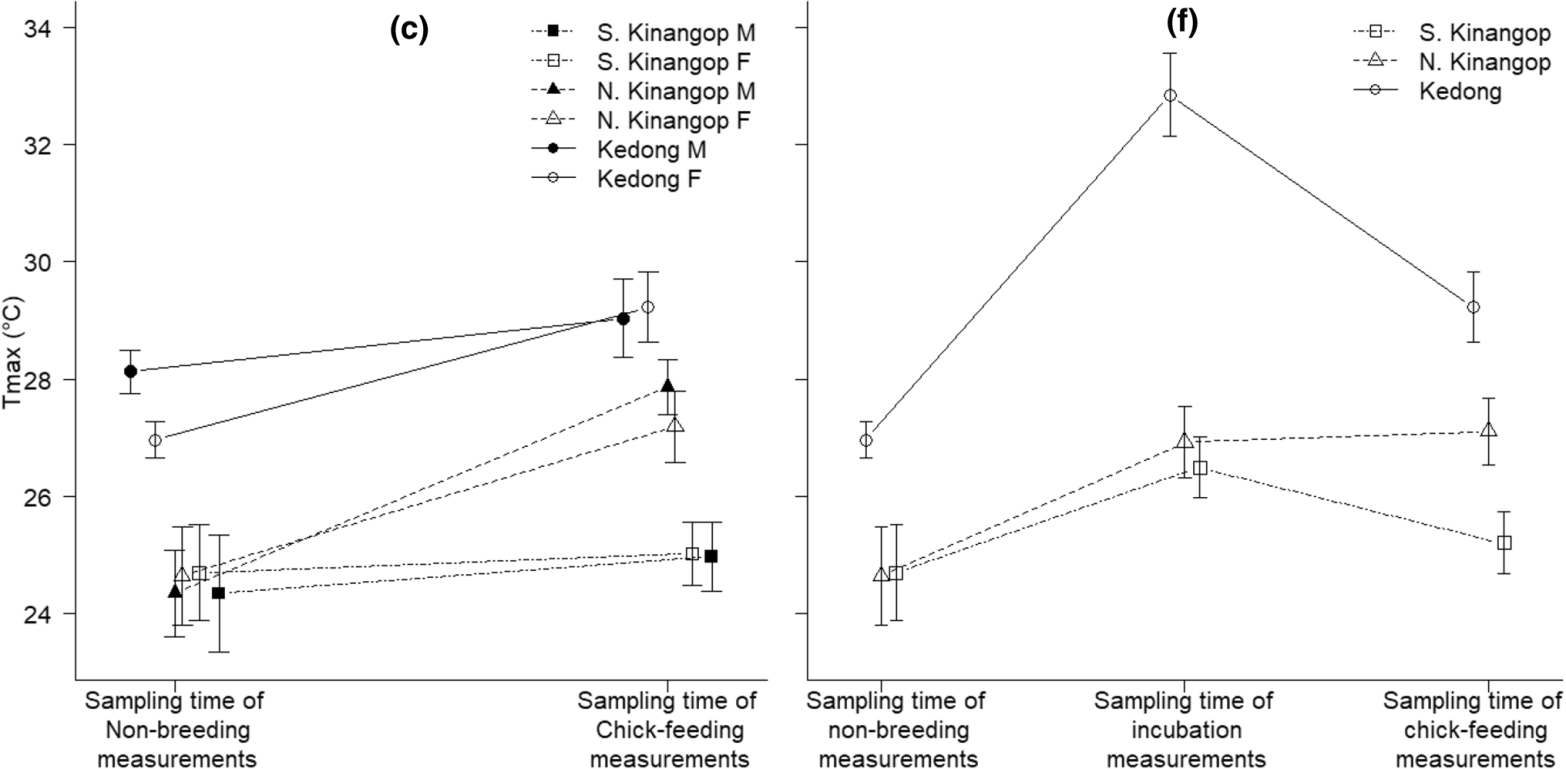
**a** Rainfall (mean ± SE, mm), **b** average minimum temperature (mean ± SE, *T*_min_, °C) and **c** average maximum temperature (mean ± SE, *T*_max_, °C) at the time of sampling non-breeding and chick-feeding male and female combined, and **d** rainfall (mean ± SE, mm), **e** average minimum temperature (mean ± SE, *T*_min_, °C) and **f** average maximum temperature (mean ± SE, *T*_max_, °C) at the time of sampling non-breeding, incubating and chick-feeding female red**-**capped larks *Calandrella cinerea* in South Kinangop (cool and wet), North Kinangop (cool and dry) and Kedong (warm and dry) in equatorial Kenya that we studied from January 2011 to March 2014

The interaction of sampling time of breeding groups × location was significant for rainfall and *T*_max_ but was borderline non-significant for *T*_min_, which instead differed according to both breeding status and location separately (Fig. 2a–c, Table 2). Post hoc tests revealed that rainfall did not differ significantly between the times that we sampled non-breeding and chick-feeding birds at any location (all *t* < 2.32, all *P* > 0.14). Among locations, the periods during which birds in the cool and wet South Kinangop fed chicks were characterized by higher rain than the periods during which birds fed chicks in the cool and dry North Kinangop (*t* = 4.29, *P* < 0.001) and in the warm and dry Kedong (*t* = 5.23, *P* < 0.001), but rainfall did not differ significantly between the cool and dry North Kinangop and the warm and dry Kedong during sampling times of chick-feeding (*t* = 0.34, *P* = 0.99). Similarly, among locations when we sampled non-breeding birds, rain was higher in the cool and wet South Kinangop (*t* = 4.01, *P* < 0.001) and in the cool and dry North Kinangop (*t* = 3.82, *P* = 0.002) compared to the warm and dry Kedong, but rain did not differ significantly between the cool and wet South and the cool and dry North Kinangop during sampling time of non-breeding birds (*t* = 0.82, *P* = 0.94).

**Table 2:** Results of models testing whether abiotic environmental factors including total monthly rain (mm), average minimum temperature (*T*_min,_ °C) and average maximum temperature (*T*_max_ °C) differ between the sampling times of chick-feeding and non-breeding male and female red**-**capped larks *Calandrella cinerea* in the cool and wet South Kinangop, the cool and dry North Kinangop and the warm and dry Kedong in equatorial Kenya

Environmental variable	Explanatory variable	DF	*F*	*P*
Rain (mm)	Sampling times of breeding groups × sex	1, 141	0.0001	0.99
	Location × sex	2, 142	0.14	0.87
	*Sampling times of breeding groups *×* location*	*2, 144*	*3.87*	***0.02***
	*Sex*	*1, 144*	*2.10*	*0.15*
*T*_min_ (°C)	Location × sex	2, 141	0.81	0.45
	Sampling times of breeding groups × sex	1, 143	1.62	0.21
	Sampling times of breeding groups × location	2, 144	2.67	0.07
	*Sampling times of breeding groups*	*1, 146*	*5.10*	***0.03***
	*location*	*2, 146*	*102.32*	** < *0.001***
	*Sex*	*1, 146*	*0.05*	*0.82*
*T*_max_ (°C)	Location × sex	2, 141	0.20	0.82
	Sampling times of breeding groups × sex	1, 143	0.76	0.39
	*Sampling times of breeding groups *×* location*	*2, 144*	*3.09*	***0.048***
	*Sex*	*1, 144*	*1.10*	*0.30*

These are *F* statistics and *P* values for parameters in the model at the last stage before parameter elimination, or in the final model, and in their order of removal. Constituent main effects of remaining interactions were not removed, and are not shown. Terms that are in the final model are in italics. Significant *P* values < 0.05 are in bold

Post hoc tests revealed that *T*_max_ was higher when we sampled chick-feeding birds compared to when we sampled non-breeding birds in the cool and dry North Kinangop (*t* = 4.43, *P* < 0.001) and in the warm and dry Kedong (*t* = 3.40, *P* = 0.01) but not in the cool and wet South Kinangop (*t *= 0.62, *P* = 0.98). We sampled chick-feeding birds in the cool and dry North Kinangop (*t* = 4.04, *P* = 0.001) and in the warm and dry Kedong (*t* = 7.41, *P* < 0.001) during periods with higher *T*_max_ compared to when we sampled birds in the cool and wet South Kinangop; the cool and dry North Kinangop and the warm and dry Kedong did not differ significantly in terms of *T*_max_ at the point of sampling chick-feeding birds (*t* = 2.72, *P* = 0.05). *T*_max_ was higher when we sampled non-breeding birds in the warm and dry Kedong than when we sampled non-breeding birds in the cool and wet South Kinangop (*t* = 4.00, *P* < 0.001) and the cool and dry North Kinangop (*t* = 5.04, *P* < 0.001). *T*_max_ when we sampled non-breeding birds did not significantly differ between the cool and wet South and the cool and dry North Kinangop (*t* = 0.11, *P* = 0.99).

When we sampled chick-feeding birds, *T*_min_ was significantly lower (mean 10.56 ± 0.29 °C, SE) compared to when we sampled non-breeding birds (mean 11.24 ± 0.27 °C, SE, Table 2). Birds in the warm and dry Kedong experienced significantly higher *T*_min_ compared to those in the cool and wet South Kinangop (*t* = 14.00, *P* < 0.001) and the cool and dry North Kinangop (*t* = 7.23, *P* < 0.001), and birds in the cool and dry North Kinangop experienced significantly higher T_min_ than those in the cool and wet South Kinangop (*t* = 6.40, *P* < 0.001).

### Immune function and body mass in non-breeding, incubating, and chick-feeding red-capped larks in three locations: female-only analyses

Breeding status had no significant effect on haptoglobin, agglutination, or lysis, but the interaction of breeding status × location again significantly affected nitric oxide (Fig. 1f–i, Table 3). Post hoc tests on the significant interaction on nitric oxide revealed one within-location effect and several among-location effects. Within the cool and wet South Kinangop, females had higher nitric oxide during incubation than during non-breeding (*t* = 3.05, *P* = 0.04). Among locations, non-breeding females in the cool and wet South Kinangop had significantly lower nitric oxide than those in the warm and dry Kedong (*t* = 3.59, *P* < 0.01). In addition, chick-feeding females in the cool and wet South Kinangop had lower nitric oxide than those in the cool and dry North Kinangop (*t* = 3.27, *P* = 0.02). All other pair-wise comparisons for nitric oxide were not significant (*t* < 2.82, *P* > 0.08). There were among-location differences for haptoglobin (Fig. 1f, Table 3) with post hoc tests revealing lower concentrations in the cool and dry North Kinangop than in the warm and dry Kedong (*t* = 3.42, P = 0.003); no other differences were significant (all *t* < 2.33, *P* > 0.06).

**Table 3:** Results of models examining variation in immune function between chick-feeding, incubating and non-breeding female (females-only dataset) red**-**capped larks *Calandrella cinerea* in the cool and wet South Kinangop, the cool and dry North Kinangop and the warm and dry Kedong in equatorial Kenya

Immune index	Response variable	DF	*F*	*P*
Haptoglobin (mg ml^−1^)	Breeding status × location	4, 91	1.01	0.41
	*Breeding status*	*2, 95*	*2.65*	*0.08*
	*Location*	*2, 95*	*6.03*	***0.003***
Nitric oxide (mmol ml^−1^)	*Breeding status *×* location*	*4, 97*	*3.13*	***0.02***
Agglutination (titre)	Breeding status × location	4, 90	1.75	0.15
	*Breeding status*	*2, 94*	*0.88*	*0.42*
	*Location*	*2, 94*	*1.14*	*0.33*
Lysis (presence/absence)	Breeding status × location	4, 92	7.47	0.11
	*Breeding status*	*2, 96*	*0.48*	*0.79*
	*Location*	*2, 96*	*2.95*	*0.24*
Mass (g)	Breeding status × location	4,113	1.35	0.26
	*Breeding status*	*2, 117*	*2.49*	*0.09*
	*Location*	*2, 117*	*19.62*	** < *0.001***

These are *F* statistics (or *X*^2^ statistic in case of lysis) and *P* values for the interaction at the last stage before parameter elimination, and for the main effects in the final model. Methodological covariates and constituent main effects of remaining interactions were not removed, and are not shown. Terms that are in the final model are in italics. Data of haptoglobin and nitric oxide were log transformed to obtain normality. *P* values < 0.05 are indicated in bold

Body mass of females differed significantly among locations (Fig. 1j, Table 3). Post hoc tests showed that females in the cool and wet South Kinangop (*t* = 5.18, *P* < 0.001) and the cool and dry North Kinangop (*t* = 5.43, *P* < 0.001) had higher body mass than those in the warm and dry Kedong, but females in the cool and wet South and the cool and dry North Kinangop did not significantly differ from each other (*t* = 0.04, *P* = 0.99).

### Rainfall and temperature at the time of sampling non-breeding, incubating, and chick-feeding red-capped larks in the three locations: female-only analyses

We found no differences in rainfall between the sampling time points of non-breeding, incubating and chick-feeding females, but rainfall did differ significantly among locations at the time points of sampling (Fig. 2d, Table 4). Post hoc tests revealed that the cool and wet South Kinangop (*t* = 4.45, *P* < 0.001) and the cool and dry North Kinangop (*t* = 2.89, *P* = 0.01) received more rain than the warm and dry Kedong. The interaction between sampling time of the breeding groups (i.e., non-breeding, incubating, chick-feeding) × location was significant for *T*_min_ and *T*_max_ (Fig. 2e, f, Table 4).

**Table 4:** Results of models testing whether abiotic environmental factors including total monthly rain (mm), average minimum temperature (*T*_min,_ °C) and average maximum temperature (*T*_max_ °C) differ between the sampling times of chick-feeding, incubating and non-breeding female red**-**capped larks *Calandrella cinerea* in the cool and wet South Kinangop, the cool and dry North Kinangop and the warm and dry Kedong in equatorial Kenya

Environmental variable	Response variable	DF	*F*	*P*
Rain (mm)	Sampling times of breeding groups × location	4, 116	1.71	0.15
	*Sampling times of breeding groups*	*2, 120*	*1.04*	*0.36*
	*Location*	*2, 120*	*9.99*	** < *0.001***
*T*_min_ (°C)	*Sampling times of breeding groups *×* location*	*4, 116*	*3.03*	***0.02***
*T*_max_ (°C)	*Sampling times of breeding groups *×* location*	*4, 116*	*4.30*	***0.003***

These are *F* statistics and *P* values for interaction at the last stage before parameter elimination, and for the main effects in the final model. Constituent main effects of remaining interactions were not removed, and are not shown. Terms that are in the final model are in italics. Significant *P* values < 0.05 are in bold

Post hoc analyses of the sampling time of the breeding groups × location interaction for *T*_min_ revealed within and among-location effects. Within the warm and dry Kedong, *T*_min_ was higher when we sampled chick-feeding females compared to when we sampled incubating females, and *T*_min_ was higher when we sampled non-breeding females compared to when we sampled incubating females (ESM Table 4a). When we sampled non-breeding females, *T*_min_ was higher in the cool and dry North Kinangop and the warm and dry Kedong compared to the cool and wet South Kinangop (ESM Table 4a). Similarly, when we sampled incubating females, *T*_min_ was higher in the cool and dry North Kinangop and the warm and dry Kedong compared to the cool and wet South Kinangop (ESM Table 4a). When we sampled chick-feeding females, *T*_min_ was higher in the warm and dry Kedong compared to the cool and wet South Kinangop and the cool and dry North Kinangop, which were marginally different from each other (ESM Table 4a). All other pair-wise comparisons were not significant (all *t* < 1.54, all *P* > 0.75).

Post hoc analyses of the sampling time of the breeding groups × location interaction for *T*_max_ also revealed within and among-location effects. Within the warm and dry Kedong, *T*_max_ was higher when we sampled incubating females compared to when we sampled chick-feeding and non-breeding females, and *T*_max_ was higher when sampling chick-feeding compared to non-breeding birds (ESM Table 4b). Among locations, *T*_max_ was higher in the warm and dry Kedong when we sampled females incubating eggs compared to both the cool and wet South and the cool and dry North Kinangop when females were incubating eggs (ESM Table 4b). Additionally, *T*_max_ was higher in the warm and dry Kedong when we sampled chick-feeding females compared to the cool and wet South Kinangop when we sampled chick-feeding females (ESM Table 4b). All other pair-wise comparisons were not significant (all *t* < 2.64, all *P* > 0.12).

## Discussion

In contrast to the prediction that immune function is suppressed by resource demanding activities such as reproduction, we found that immune indices of year-round breeding equatorial red-capped larks did not differ between breeding (incubation and chick-feeding) and non-breeding birds. This finding was mostly consistent across the three different equatorial populations. There were only two exceptions: higher concentrations of nitric oxide (NOx) in chick-feeding birds compared to non-breeding ones in the cool and dry North Kinangop and in incubating females compared to non-breeding ones in the cool and wet South Kinangop. These reproduction-associated increases are the reverse of our expectation that reproduction would lead to reductions in immune indices as a result of trade-offs. Body mass also did not differ between breeding and non-breeding birds in any of the populations, suggesting that birds do not trade-off their body mass for reproduction. Based on our previous work, we had expected that environmental conditions would not differ between the times we sampled breeding and non-breeding birds in each location (Ndithia et al. 2017a). However, we actually found differences between chick-feeding sampling time points and non-breeding sampling time points in two locations for *T*_max_ and three for *T*_min_. In contrast to the general absence of differences in immune indices between breeding and non-breeding birds, we found significant differences for all four immune indices among locations with different climates. The exact nature of these effects depended on the immune index and breeding status, but not sex. Thus, we propose that in tropical equatorial birds immunological variation is better explained by climate-induced environmental conditions (that uses location as proxy for different climatic conditions), which are typically localized and unpredictable, than by breeding status. Our findings raise questions about the mechanisms underlying this link.

The absence of the predicted downregulation of immune function during breeding (i.e., incubation and chick-feeding compared to non-breeding) in all locations might point to an evolutionary link between the pace of life, specifically clutch size, and immune function. Red-capped larks, a tropical equatorial and long lived species, exhibits a slow pace of life and produces clutches of only two eggs. Two-egg clutches are relatively small compared to those of bird species living in the temperate and arctic zones. A small clutch size may allow red-capped larks to simultaneously maintain both reproduction and immune function. Long lived birds with a slower pace of life are associated with well-developed immune defences (Martin et al. 2006; Lee et al. 2008) and are known to favour investments that increase survival, including immune defenses, even under challenging conditions such as reproduction (Tella et al. 2002; Ardia 2005; Lee 2006; Lee et al. 2008; Tieleman et al. 2019; Schultz et al. 2020). On the other hand, birds in mid-to-high latitudes are short-lived and have a faster pace of life and supposedly invest in reproduction (large clutch size) at the expense of self-maintenance (Martin et al. 2006; Lee et al. 2008). It remains to be determined whether the elevated NOx concentration, as seen in some reproductively active red-capped larks, results from increased work load (from reproduction), indicates some breeding birds are of higher quality than others (i.e., can defend themselves well and reproduce; see Saino et al. 1997), or implies birds in certain physiological condition (e.g., low NOx) skipped breeding.

The three populations living in climatically distinct locations differed in their immune indices, but no one index consistently differed in the same way among locations. The finding that among-location differences in immune function vary according to breeding status suggests that immune function does not simply reflect overall long-term (e.g., annual) patterns in climate but also at least partly responds to current weather conditions, despite their unpredictability (Ndithia et al. 2017a). Although on average rainfall, *T*_min_, and *T*_max_ differed among locations, these differences may not be present or have the same magnitude of effect in all months of the year or during different breeding statuses. (Ndithia et al. 2017a).

Our findings suggest that different immune indices were differently influenced by environmental conditions using location as proxy for different climatic conditions. Temperature (Shephard et al. 1998; Bowden et al. 2007; Schultz et al. 2020) and rainfall (Rubenstein et al. 2008; Schultz et al. 2020) have been shown to influence different components of immune function. Moreover, temperature (Watts et al. 1987; Demas and Nelson 1998; Altizer et al. 2006; Lowen et al. 2007) and rainfall (Bicout and Sabatier 2004; Tieleman et al. 2019) can influence both the broader geographical patterns and the short-term local dynamics of pathogens and diseases, which would be expected to drive immunological variation (Christe et al. 2001; Møller et al. 2003; Horrocks et al. 2012b; Tieleman et al. 2019; Schultz et al. 2020). Unpredictable changes in environmental conditions (e.g., disease prevalence, unpredictable weather patterns), may cause perturbations in animals to levels that may have negative consequences on their physiology, e.g., mobilization of the immune system, and in more severe conditions, immunosuppression (McEwen and Wingfield 2003; Romero et al. 2009). We propose that organisms living in different locations develop environment-specific immune strategies that are shaped directly and indirectly by prevailing (or otherwise dominant) environmental conditions. An alternative hypothesis that resource availability influences immune function via trade-offs appears to be less relevant in the current study system, where food is available independent of rain or temperature (Ndithia et al. 2017a; Mwangi et al. 2018).

The unexpected differences in *T*_max_ and *T*_min_ between chick-feeding and non-breeding sampling time points suggest that red-capped lark parents utilized environmental conditions differently in the different locations for reproductive success. *T*_max_ and *T*_min_ may have influenced reproduction through their influence on food availability, as other studies in tropical systems have suggested (Intachat et al. 2001; Michel and Letourmy 2007). Environmental conditions did not influence the occurrence or the intensity of reproduction as measured by presence of nests (Ndithia et al. 2017a). However, rainfall affected nestling growth in the warm and dry Kedong, either directly (e.g., through regulation of gonad sizes or reproductive hormones, Hau 2001) or indirectly (e.g., via effects on food availability). Parents in the different locations utilized food differently for reproduction, leading to differences in nestling growth rate among locations (Ndithia et al. 2017b). The seeming disparity between these sets of findings partially arises from different measures of reproduction that we used in the different papers. Ndithia et al. (2017a) tested for the relationship between reproduction and environmental conditions using data from all found nests (which were at various stages, including nest construction, incubation, and chick-feeding), some of which were unsuccessful. Conversely, Ndithia et al. (2017b) tested for the effects of environmental conditions on nestling growth using reproductive data at nestling stage with nests that were successful up to the point of (near)-fledging of the nestlings. The current study used data on egg incubation and chick-feeding in adult birds, hence beyond the point of nest construction and egg-laying (Ndithia et al. 2017a). It also involves fewer nests than Ndithia et al. (2017a) and hence possibly the difference in environmental conditions between sampling times has occurred by chance.

In conclusion, the location where a bird lives, both in the global and the local sense, seems to matter in terms of immunological variation. Temperate and arctic zone birds live in environments characterized by predictable seasonal changes and presumably exhibit trade-offs between reproduction and immune function. In contrast, tropical equatorial birds, like the red-capped larks we studied, face unpredictable environmental conditions and maintain their immune function, particularly innate immune function, during energetically demanding life cycle stages, including reproduction. Furthermore, within the equatorial tropics, red-capped larks living in environments with different local climates seem to exhibit different immune strategies that are influenced by current environmental conditions. Future studies should focus on the role of different environmental conditions on the spatio-temporal dynamics of pathogens and parasites and, presumably, the influence of these dynamics on immunological variation in birds living near the equator (and at other latitudes). In addition, efforts to understand such immunological variation in an ecological framework should also focus on the mechanisms that might allow some birds to maintain, or even increase, defences while engaged in costly activities like reproduction.

## Supplementary Information

Below is the link to the electronic supplementary material.

Supplementary file1 (DOCX 15 kb)

Supplementary file2 (PDF 299 kb)

Supplementary file3 (PDF 100 kb)

Supplementary file4 (PDF 531 kb)

Supplementary file5 (DOCX 19 kb)

## Declarations

### Welfare of animals used for research

The National Museums of Kenya, the authority over bird research in Kenya, approved this work. We (Irene Tieleman, Henry Kamau Ndithia, Peter Kinyanjui Gachigi, Paul Maina Kimani and Abraham Mwangi Kuria) are all skilled bird ringers, and we captured and handled birds in adherence to animal welfare and the standard operating protocols of the National Museums of Kenya. Therefore, all applicable institutional and/or national guidelines for the care and use of animals were followed.
