# Supplementary material for: Immune function differs among tropical environments but is not downregulated during reproduction in three year-round breeding equatorial lark populations
Source: Oecologia. 2021 Oct 12;197(3):599–614. doi: 10.1007/s00442-021-05052-0 (PMC8585810; doi:10.1007/s00442-021-05052-0)
Supplement: Supplementary file 1 — Supplementary file1 (DOCX 15 kb) [file 442_2021_5052_MOESM1_ESM.docx]

Immune function differs among tropical environments but is not downregulated during reproduction in three year-round breeding equatorial lark populations

Submitted to Oecologia

Henry K. Ndithia^1, 2,*^, Kevin D. Matson^3^, Muchane Muchai^1, 4^, B. Irene Tieleman^2^

^1^Ornithology Section, Department of Zoology, National Museums of Kenya, P.O. Box 40658 – 00100 GPO, Nairobi, Kenya

^2^Groningen Institute for Evolutionary Life Sciences, University of Groningen, P.O. Box 11103, 9700 CC Groningen, The Netherlands;

^3^Resource Ecology Group, Department of Environmental Sciences, Wageningen University, Droevendaalsesteegh 3a, 6708 PB Wageningen, The Netherlands

^4^Department of Clinical Studies (Wildlife and Conservation), College of Agriculture and Veterinary Sciences, University of Nairobi. Box 30197**-**00100, Nairobi, Kenya

*Corresponding author:

Email: hndithia@gmail.com

ESM Table 1b. Monthly climatic characteristics of our three Kenyan study locations (cool and wet South Kinangop, cool and dry North Kinangop, and warm and dry Kedong) where we investigated the role of reproduction and environmental on the variation in immune function in red-capped larks *Calandrella cinerea* from January 2011 to March 2014

|  | **South Kinangop**  **(cool and wet)** | | | **North Kinangop**  **(Cool and dry)** | | | **Kedong**  **(Warm and dry)** | | |
| --- | --- | --- | --- | --- | --- | --- | --- | --- | --- |
| **Month** | **rain (mm)** | **T_max_ (**°**C)** | **T_min_ (**°**C)** | **rain (mm)** | **T_max_ (**°**C)** | **T_min_ (**°**C)** | **rain (mm)** | **T_max_ (**°**C)** | **T_min_ (**°**C)** |
| Jan-11 | 18.6 | 27.1 | 3.1 | 8.4 | 27.7 | 6.5 | 8.4 | 27.7 | 15.2 |
| Feb-11 | 32.4 | 27.9 | 3.8 | 14.0 | 29.8 | 7.2 | 3.1 | 29.8 | 13.0 |
| Mar-11 | 120.0 | 26.8 | 4.9 | 89.4 | 27.5 | 9.4 | 69.5 | 29.2 | 15.7 |
| Apr-11 | 63.1 | 26.2 | 5.2 | 39.0 | 26.8 | 11.2 | 5.8 | 29.9 | 12.6 |
| May-11 | 35.2 | 23.8 | 5.3 | 14.9 | 26.6 | 11.5 | 16.1 | 27.5 | 10.9 |
| Jun-11 | 103.7 | 22.8 | 5.0 | 40.9 | 24.6 | 10.2 | 37.9 | 27.5 | 10.6 |
| Jul-11 | 63.6 | 23.9 | 3.8 | 17.3 | 25.4 | 8.6 | 13.7 | 28.3 | 8.0 |
| Aug-11 | 53.3 | 24.0 | 4.5 | 74.9 | 26.0 | 10.3 | 46.6 | 25.9 | 10.3 |
| Sep-11 | 87.2 | 23.2 | 5.0 | 70.9 | 23.2 | 12.1 | 42.9 | 28.0 | 10.3 |
| Oct-11 | 84.3 | 24.7 | 6.1 | 70.6 | 25.6 | 8.6 | 66.4 | 28.9 | 10.8 |
| Nov-11 | 233.9 | 24.4 | 7.2 | 85.5 | 26.7 | 10.3 | 86.2 | 30.2 | 12.7 |
| Dec-11 | 98.7 | 25.2 | 5.9 | 49.6 | 27.0 | 9.0 | 123.1 | 29.2 | 11.4 |
| Jan-12 | 19.4 | 29.1 | 4.6 | 1.2 | 30.5 | 3.0 | 0.0 | 34.9 | 6.2 |
| Feb-12 | 3.4 | 28.0 | 3.0 | 9.2 | 29.8 | 4.2 | 20.2 | 31.8 | 10.1 |
| Mar-12 | 13.5 | 27.2 | 4.3 | 15.7 | 28.8 | 4.6 | 4.2 | 33.5 | 8.4 |
| Apr-12 | 203.5 | 24.2 | 7.4 | 97.5 | 26.1 | 10.7 | 117.7 | 28.9 | 12.6 |
| May-12 | 217.6 | 21.9 | 7.1 | 104.1 | 23.2 | 11.8 | 65.3 | 26.2 | 11.5 |
| Jun-12 | 82.2 | 21.2 | 5.5 | 30.1 | 22.6 | 10.3 | 10.9 | 25.3 | 9.8 |
| Jul-12 | 66.9 | 22.0 | 6.8 | 14.4 | 22.5 | 8.6 | 32.7 | 25.5 | 10.3 |
| Aug-12 | 100.1 | 21.3 | 5.3 | 49.4 | 24.7 | 7.6 | 15.5 | 27.0 | 9.3 |
| Sep-12 | 61.6 | 22.6 | 5.0 | 71.9 | 23.7 | 7.3 | 17.9 | 28.8 | 8.6 |
| Oct-12 | 63.2 | 23.6 | 5.7 | 90.9 | 26.3 | 8.3 | 33.8 | 29.7 | 10.4 |
| Nov-12 | 77.2 | 24.9 | 5.7 | 58.6 | 25.7 | 9.8 | 9.4 | 30.4 | 9.4 |
| Dec-12 | 119.8 | 25.4 | 5.5 | 2.5 | 27.7 | 7.2 | 3.7 | 31.9 | 10.2 |
| Jan-13 | 49.9 | 26.0 | 6.5 | 0.0 | 30.2 | 5.9 | 17.1 | 32.4 | 9.5 |
| Feb-13 | 1.0 | 27.0 | 6.1 | 0.0 | 26.8 | 9.9 | 16.4 | 32.6 | 7.9 |
| Mar-13 | 79.4 | 27.7 | 6.4 | 73.2 | 27.0 | 10.3 | 38.7 | 30.5 | 11.3 |
| Apr-13 | 309.0 | 24.8 | 8.2 | 155.3 | 24.0 | 12.9 | 95.1 | 26.7 | 13.4 |
| May-13 | 74.3 | 24.0 | 5.7 | 42.5 | 24.1 | 8.2 | 9.7 | 26.2 | 11.9 |
| Jun-13 | 23.3 | 23.7 | 4.7 | 62.0 | 23.4 | 7.1 | 0.0 | 26.9 | 11.8 |
| Jul-13 | 40.0 | 24.6 | 5.1 | 56.3 | 22.6 | 8.9 | 0.0 | 28.5 | 9.5 |
| Aug-13 | 31.5 | 22.7 | 5.6 | 19.1 | 23.4 | 10.9 | 0.0 | 26.7 | 10.3 |
| Sep-13 | 18.5 | 23.7 | 5.3 | 46.9 | 24.2 | 8.8 | 0.5 | 27.7 | 12.6 |
| Oct-13 | 20.3 | 24.7 | 4.3 | 45.7 | 24.3 | 8.3 | 0.0 | 28.1 | 9.7 |
| Nov-13 | 38.7 | 23.3 | 6.4 | 50.3 | 22.1 | 13.7 | 0.0 | 28.8 | 6.3 |
| Dec-13 | 141.2 | 24.1 | 6.1 | 66.9 | 22.7 | 13.2 | 46.8 | 25.7 | 12.2 |
| Jan-14 | 18.9 | 30.0 | 4.4 | 6.6 | 23.8 | 7.3 | 40.0 | 26.2 | 11.1 |
| Feb-14 | 0.0 | 26.7 | 6.1 | 30.0 | 23.5 | 8.7 | 153.2 | 25.5 | 12.1 |
| Mar-14 | 0.0 | 27.0 | 5.2 | 15.0 | 23.1 | 9.0 | 75.2 | 24.8 | 11.9 |
