# Supplementary material for: Immune function differs among tropical environments but is not downregulated during reproduction in three year-round breeding equatorial lark populations
Source: Oecologia. 2021 Oct 12;197(3):599–614. doi: 10.1007/s00442-021-05052-0 (PMC8585810; doi:10.1007/s00442-021-05052-0)
Supplement: Supplementary file 2 — Supplementary file2 (PDF 299 kb) [file 442_2021_5052_MOESM2_ESM.pdf]

Immune function differs among tropical environments but is not downregulated during reproduction in three year-round breeding equatorial lark populations

Submitted to Oecologia

Henry K. Ndithia[1, 2,*], Kevin D. Matson[3], Muchane Muchai[1, 4], B. Irene Tieleman[2]

[1]Ornithology Section, Department of Zoology, National Museums of Kenya, P.O. Box 40658 – 00100 GPO, Nairobi, Kenya

[2]Groningen Institute for Evolutionary Life Sciences, University of Groningen, P.O. Box 11103, 9700 CC Groningen, The Netherlands;

[3]Resource Ecology Group, Department of Environmental Sciences, Wageningen University, Droevendaalsesteegh 3a, 6708 PB Wageningen, The Netherlands

[4]Department of Clinical Studies (Wildlife and Conservation), College of Agriculture and Veterinary Sciences, University of Nairobi. Box 30197-00100, Nairobi, Kenya

*Corresponding author:

Email: hndithia@gmail.com

ESM Table 2. Sample sizes of non-breeding and chick-feeding females and males (sexes-combined dataset), and sample sizes of non-breeding, incubating and chick-feeding females (females-only dataset) of red-capped larks *Calandrella cinerea* during our study of the influence of reproduction and the environment on the variation in immune function in South Kinangop (cool and wet), North Kinangop (cool and dry) and Kedong (warm and dry) in equatorial Kenya from January 2011 to March 2014. While testing for effects of reproduction and the environment on immune function, we separately analysed using similar approaches, two data sets, sexes combined and females only, because only females in this species incubate eggs

| **Parameter** | **breeding status** | **sex** | **South Kinangop (cool and wet)** | **North Kinangop (cool and dry)** | **Kedong (Warm and dry)** |
|---|---|---|---|---|---|
| Immune function of males and females | non-breeding | f | 4 | 5 | 22 |
| | | m | 5 | 9 | 21 |
| | chick-feeding | f | 12 | 13 | 21 |
| | | m | 11 | 10 | 15 |
| Environmental variables of males and females | non-breeding | f | 4 | 5 | 24 |
| | | m | 5 | 10 | 22 |
| | chick-feeding | f | 11 | 13 | 20 |
| | | m | 12 | 9 | 16 |
| Immune function | non-breeding | f | 4 | 5 | 22 |

| | | | | | |
|---|---|---|---|---|---|
| of female | incubating | f | 10 | 10 | 19 |
| | chick-feeding | f | 12 | 13 | 21 |
| Environmental | non-breeding | f | 4 | 5 | 24 |
| variables | incubating | f | 12 | 13 | 21 |
| of female | chick-feeding | f | 12 | 14 | 20 |
