# Supplementary material for: Immune function differs among tropical environments but is not downregulated during reproduction in three year-round breeding equatorial lark populations
Source: Oecologia. 2021 Oct 12;197(3):599–614. doi: 10.1007/s00442-021-05052-0 (PMC8585810; doi:10.1007/s00442-021-05052-0)
Supplement: Supplementary file 3 — Supplementary file3 (PDF 100 kb) [file 442_2021_5052_MOESM3_ESM.pdf]

Immune function differs among tropical environments but is not downregulated during reproduction in three year-round breeding equatorial lark populations

Submitted to Oecologia

Henry K. Ndithia[1, 2,*], Kevin D. Matson[3], Muchane Muchai[1, 4], B. Irene Tieleman[2]

[1]Ornithology Section, Department of Zoology, National Museums of Kenya, P.O. Box 40658 – 00100 GPO, Nairobi, Kenya

[2]Groningen Institute for Evolutionary Life Sciences, University of Groningen, P.O. Box 11103, 9700 CC Groningen, The Netherlands;

[3]Resource Ecology Group, Department of Environmental Sciences, Wageningen University, Droevendaalsesteegh 3a, 6708 PB Wageningen, The Netherlands

[4]Department of Clinical Studies (Wildlife and Conservation), College of Agriculture and Veterinary Sciences, University of Nairobi. Box 30197-00100, Nairobi, Kenya

*Corresponding author:
Email: hndithia@gmail.com

ESM Table 3a. Coefficient estimates, standard error, t and P values for models with immune indices (haptoglobin, nitric oxide, agglutination and lysis), and body mass as dependent variables and the different explanatory variables in the study investigating i) the effect of breeding (non-breeding, chick-feeding), location, sex and their two way interaction in the case of sexes-combined dataset, and ii) the effect of breeding (non-breeding, incubating and chick-feeding), location and their interaction in the case of female-only dataset, on immune function that we carried out from January 2011 to March 2014 in three climatically-distinct locations: cool and wet South Kinangop (SK), cool and dry North Kinangop (NK) and warm and dry Kedong (KE). Significant P values are in bold

i)

Haptoglobin: male and female

Model	Explanatory variables	Estimate	SE	t	P
lm(log10(hp)~Location*breedingStatus+Location*sex+breedingStatus*sex+plasma sample age+redness_450) (starting model before elimination of interaction Location:breedingStatus)	(Intercept)	-0.89	0.15	-6.04	**<0.001**
	LocationNK	-0.38	0.16	-2.48	**0.01**
	LocationSK	-0.06	0.17	-0.36	0.72
	breedingStatusChick-feeding	0.19	0.12	1.56	0.12
	sexM	0.05	0.11	0.43	0.67
	sample_age	0.001	<0.001	3.70	**<0.001**
	redness_450	1.11	0.31	3.65	**<0.001**
	LocationNK:breedingStatusChick-feeding	0.08	0.16	0.49	0.62
	LocationSK:breedingStatusChick-feeding	-0.15	0.18	-0.84	0.40
	LocationNK:sexM	0.43	0.16	2.63	**0.01**
	LocationSK:sexM	0.28	0.17	1.61	0.11
	breedingStatusChick-feeding:sexM	-0.18	0.14	-1.33	0.19
lm(log10(hp)~Location*sex+breedingStatus*sex+sample_age+redness_450)	(Intercept)	-0.89	0.15	-6.09	**<0.001**
	LocationNK	-0.32	0.12	-2.79	**0.01**
	LocationSK	-0.17	0.12	-1.34	0.18
	breedingStatusChick-feeding	0.18	0.11	1.70	0.09
	sexM	0.05	0.11	0.41	0.68

(model after elimination of interaction Location:breedingStatus)	Plasma sample age	0.001	<0.001	3.83	**<0.001**
	Plasma sample redness_450	1.09	0.30	3.60	**<0.001**
	LocationNK:sexM	0.41	0.16	2.57	**0.01**
	LocationSK:sexM	0.29	0.17	1.67	0.10
	breedingStatusChick-feeding:sexM	-0.18	0.14	-1.32	0.19
lm(log10(hp)~Location*sex+plasma sample age+redness_450) (Final model: model after elimination of interaction breedingStatus:sex)	(Intercept)	-0.87	0.15	-5.94	**<0.001**
	LocationNK	-0.30	0.12	-2.62	**0.01**
	LocationSK	-0.14	0.12	-1.16	0.25
	breedingStatusChick-feeding	0.09	0.08	1.09	0.28
	sexM	-0.03	0.09	-0.37	0.71
	plasma sample age	0.001	0.00	3.87	**<0.001**
	redness_450	1.14	0.30	3.76	**<0.001**
	LocationNK:sexM	0.38	0.16	2.40	**0.02**
	LocationSK:sexM	0.25	0.17	1.45	0.15
**Nitric oxide: male and female**					
lm(log10(Nox)~Location*breedingStatus+Location*sex+bredingStatus*sex) (starting model before elimination of interaction breedingStatus:sex)	(Intercept)	-2.02	0.08	-25.62	**<0.001**
	LocationNK	-0.25	0.15	-1.72	0.09
	LocationSK	-0.71	0.17	-4.17	**<0.001**
	breedingStatusChick-feeding	-0.22	0.10	-2.08	**0.04**
	sexM	-0.06	0.10	-0.59	0.56
	LocationNK:breedingStatusChick-feeding	0.57	0.15	3.68	**<0.001**
	LocationSK:breedingStatusChick-feeding	0.47	0.17	2.73	**0.01**
	LocationNK:sexM	-0.11	0.15	-0.73	0.47
	LocationSK:sexM	0.11	0.16	0.69	0.49
	breedingStatusChick-feeding:sexM	0.08	0.13	0.61	0.54
lm(log10(Nox)~Location*breedingStatus+Location*sex) (model after elimination of interaction breedingStatus:sex)	(Intercept)	-2.04	0.07	-28.18	**<0.001**
	LocationNK	-0.26	0.14	-1.82	**0.07**
	LocationSK	-0.72	0.17	-4.34	**<0.001**
	breedingStatusChick-feeding	-0.18	0.08	-2.12	**0.04**
	sexM	-0.02	0.08	-0.29	0.77
	LocationNK:breedingStatusChick-feeding	0.57	0.15	3.72	**<0.001**
	LocationSK:breedingStatusChick-feeding	0.48	0.17	2.81	**0.01**
	LocationNK:sexM	-0.10	0.15	-0.66	0.51
	LocationSK:sexM	0.13	0.16	0.83	0.41

lm(log10(Nox)~Location*breedingStatus) (Final model: model after elimination of interaction Location:sex)	(Intercept)	-2.04	0.07	-30.77	**<0.001**
	LocationNK	-0.32	0.11	-2.83	**0.01**
	LocationSK	-0.64	0.14	-4.66	**<0.001**
	breedingStatusChick-feeding	-0.18	0.08	-2.13	**0.04**
	sexM	-0.02	0.06	-0.34	0.73
	LocationNK:breedingStatusChick-feeding	0.59	0.15	3.95	**<0.001**
	LocationSK:breedingStatusChick-feeding	0.46	0.17	2.73	**0.01**
**Agglutination: male and female**					
lm(AvAgglut2~Location*breedingStatus+Location*sex+breedingStatus*sex+ plasma sample age) (starting model before elimination-of interaction Location:breedingStatus)	(Intercept)	8.20	0.89	9.20	**<0.001**
	LocationNK	-0.85	1.17	-0.73	0.47
	LocationSK	-0.25	1.22	-0.20	0.84
	breedingStatusChick-feeding	-0.61	0.76	-0.80	0.42
	sexM	-0.35	0.77	-0.46	0.64
	sample_age	-0.003	0.001	-3.74	**<0.001**
	LocationNK:breedingStatusChick-feeding	0.32	1.19	0.27	0.79
	LocationSK:breedingStatusChick-feeding	-0.56	1.29	-0.44	0.66
	LocationNK:sexM	-1.14	1.18	-0.97	0.33
	LocationSK:sexM	-1.20	1.21	-0.99	0.32
	breedingStatusChick-feeding:sexM	0.96	0.98	0.98	0.33
lm(AvAgglut2~Location*sex+breedingStatus*sex+ plasma sample age) (model after elimination of interaction Location:breedingStatus)	(Intercept)	8.20	0.87	9.44	**<0.001**
	LocationNK	-0.60	0.83	-0.72	0.47
	LocationSK	-0.66	0.82	-0.80	0.43
	breedingStatusChick-feeding	-0.65	0.67	-0.98	0.33
	sexM	-0.37	0.76	-0.49	0.62
	sample_age	-0.003	0.001	-3.74	**<0.001**
	LocationNK:sexM	-1.22	1.15	-1.06	0.29
	LocationSK:sexM	-1.20	1.20	-1.00	0.32
	breedingStatusChick-feeding:sexM	1.00	0.96	1.04	0.30
lm(AvAgglut2~breedingStatus*sex+plasma sample age) (Final model: model after elimination of	(Intercept)	8.28	0.87	9.57	**<0.001**
	LocationNK	-1.21	0.58	-2.09	**0.04**
	LocationSK	-1.19	0.61	-1.96	0.05
	breedingStatusChick-feeding	-0.51	0.66	-0.78	0.44
	sexM	-0.77	0.69	-1.12	0.27

interaction Location:sex)	sample_age	-0.003	0.001	-3.65	**<0.001**
	breedingStatusChick-feeding:sexM	0.70	0.93	0.75	0.45
lm(AvAgglut2~Location+breedingStatus+sex+ plasma sample age) (Final model: model after elimination of interaction breedingStatus:sex)	(Intercept)	8.05	0.81	9.96	**<0.001**
	LocationNK	-1.23	0.58	-2.13	**0.04**
	LocationSK	-1.18	0.61	-1.94	0.05
	breedingStatusChick-feeding	-0.18	0.49	-0.37	0.71
	sexM	-0.39	0.46	-0.83	0.41
	sample_age	-0.003	0.001	-3.61	**<0.001**
**Lysis: male and female**					
glm(AvLysis2~Location+breedingStatus+sex+Location:breedingStatus+Location:sex+breedingStatus:sex+plasma sample age, binomial) (starting model before elimination of interaction breedingStatus:sex)	intercept	4.24	1.12	3.80	**<0.001**
	LocationNK	-2.06	1.30	-1.59	0.11
	LocationSK	-2.90	1.32	-2.20	**0.03**
	breedingStatusChick-feeding	-1.45	0.77	-1.88	0.06
	sexM	-0.55	0.80	-0.68	0.49
	sample_age	-0.005	0.001	-4.82	**<0.001**
	LocationNK:breedingStatusChick-feeding	2.55	1.26	2.02	**0.04**
	LocationSK:breedingStatusChick-feeding	4.23	1.37	3.09	**<0.001**
	LocationNK:sexM	0.25	1.18	0.22	0.83
	LocationSK:sexM	1.11	1.17	0.95	0.34
	breedingStatusChick-feeding:sexM	0.40	1.04	0.39	0.70
glm(AvLysis2~Location+breedingStatus+sex+Location:breedingStatus+Location:sex+ plasma sample age, binomial) (model after elimination of interaction breedingStatus:sex)	(Intercept)	4.13	1.06	3.88	**<0.001**
	LocationNK	-2.22	1.25	-1.78	0.08
	LocationSK	-2.98	1.31	-2.27	**0.02**
	breedingStatusChick-feeding	-1.31	0.68	-1.93	0.05
	sexM	-0.40	0.69	-0.57	0.57
	sample_age	-0.005	0.001	-4.85	**<0.001**
	LocationNK:breedingStatusChick-feeding	2.68	1.23	2.17	**0.03**
	LocationSK:breedingStatusChick-feeding	4.26	1.38	3.10	**<0.001**
	LocationNK:sexM	0.41	1.12	0.37	0.71
	LocationSK:sexM	1.28	1.08	1.18	0.24
glm(AvLysis~Location+breedingStatus+sex+Location:bree	(Intercept)	3.90	1.03	3.78	**<0.001**
	LocationNK	-2.07	1.00	-2.08	**0.04**
	LocationSK	-2.36	1.18	-1.99	0.05

dingStatus+ plasma sample age, binomial)<br><br>(Final model: model after elimination of interaction Location:sex)	breedingStatusChick-feeding	-1.26	0.67	-1.87	0.06
	sexM	0.10	0.45	0.22	0.82
	sample_age	-0.005	0.001	-4.83	**<0.001**
	LocationNK:breedingStatus Chick-feeding	2.66	1.20	2.21	**0.03**
	LocationSK:breedingStatus Chick-feeding	4.16	1.35	3.07	**<0.001**
**Mass: male and female**					
lm(mass~Location+ breedingStatus+sex +Location:breeding Status+Location:sex +breedingStatus:sex)<br><br>(starting model before elimination of interaction Location:sex)	(Intercept)	23.43	0.34	69.43	< **0.001**
	LocationNK	2.97	0.68	4.33	< **0.001**
	LocationSK	2.49	0.76	3.30	< **0.001**
	breedingStatusChick-feeding	0.37	0.48	0.77	0.44
	sexM	0.25	0.47	0.54	0.59
	LocationNK:breedingStatus Chick-feeding	-1.88	0.72	-2.61	**0.01**
	LocationSK:breedingStatus Chick-feeding	-1.31	0.80	-1.64	**0.10**
	LocationNK:sexM	-0.71	0.72	-0.99	0.32
	LocationSK:sexM	-0.42	0.77	-0.54	0.59
	breedingStatusChick-feeding:sexM	0.90	0.60	1.51	**0.13**
lm(mass~Location+ breedingStatus+sex +Location:breeding Status+breedingStat us:sex)<br><br>(model after elimination of interaction Location:sex)	(Intercept)	23.52	0.32	72.71	< **0.001**
	LocationNK	2.54	0.52	4.85	< **0.001**
	LocationSK	2.28	0.63	3.61	< **0.001**
	breedingStatusChick-feeding	0.42	0.47	0.88	0.38
	sexM	0.05	0.42	0.13	0.90
	LocationNK:breedingStatus Chick-feeding	-1.75	0.70	-2.49	**0.01**
	LocationSK:breedingStatus Chick-feeding	-1.29	0.80	-1.62	0.11
	breedingStatusChick-feeding:sexM	0.79	0.58	1.35	0.18
lm(mass~Location+ breedingStatus+sex +Location:breeding Status+)<br><br>(Final model: model after elimination of interaction breedingStatus:sex)	(Intercept)	23.33	0.29	79.85	< **0.001**
	LocationNK	2.45	0.52	4.71	< **0.001**
	LocationSK	2.24	0.63	3.54	< **0.001**
	breedingStatusChick-feeding	0.77	0.39	1.95	0.05
	sexM	0.47	0.29	1.63	0.11
	LocationNK:breedingStatus Chick-feeding	-1.67	0.70	-2.38	**0.02**
	LocationSK:breedingStatus Chick-feeding	-1.23	0.80	-1.54	0.13

ii)

Haptoglobin: females only

Model	Explanatory variables	Estimate	SE	t	P
lm(log10(hp)~Location+breedingStatus +Location:breedingStatus) (starting model before elimination of interaction Location:breedingStatus)	(Intercept)	-0.24	0.10	-2.48	**0.01**
	LocationNK	-0.51	0.21	-2.39	**0.02**
	LocationSK	0.05	0.23	0.20	0.85
	breedingStatusBincubating	0.05	0.14	0.38	0.71
	breedingStatusChick-feeding	0.06	0.15	0.40	0.69
	LocationNK:breedingStatus Bincubating	0.08	0.27	0.28	0.78
	LocationSK:breedingStatus Bincubating	-0.04	0.29	-0.14	0.89
	LocationNK:breedingStatus Chick-feeding	0.36	0.27	1.33	0.19
	LocationSK:breedingStatus Chick-feeding	-0.21	0.29	-0.73	0.47
lm(log10(hp)~Location+breedingStatus)) (Final model: model after elimination of interaction Location:breedingStatus)	(Intercept)	-0.26	0.09	-3.02	**0.003**
	LocationNK	-0.33	0.10	-3.21	**0.002**
	LocationSK	-0.07	0.11	-0.65	0.52
	breedingStatusBincubating	0.06	0.11	0.54	0.59
	breedingStatusChick-feeding	0.11	0.11	0.97	0.33

Nitric oxide: females only

Model	Explanatory variables	Estimate	SE	t	P
lm(log10(Nox)~Location+breedingStatus+Location:breedingStatus) (starting and final model: interaction Location:breedingStatus significant)	(Intercept)	-2.05	0.08	-25.61	< **0.001**
	LocationNK	-0.06	0.17	-0.36	0.72
	LocationSK	-0.76	0.21	-3.59	**0.001**
	breedingStatusBincubating	-0.07	0.11	-0.59	0.55
	breedingStatusChick-feeding	-0.16	0.11	-1.41	0.16
	LocationNK:breedingStatus Bincubating	0.04	0.22	0.17	0.87
	LocationSK:breedingStatus Bincubating	0.75	0.25	2.99	**0.003**
	LocationNK:breedingStatus Chick-feeding	0.29	0.21	1.37	0.17
	LocationSK:breedingStatus Chick-feeding	0.52	0.25	2.08	**0.04**

Agglutination: females only

lm(AvAgglut~Location*breedingStatus + plasma sample age, data=AggF)<br><br>(starting model before elimination of interaction Location:breedingStatus)	(Intercept)	8.03	1.20	6.70	**<0.001**
	LocationNK	1.76	1.56	1.13	0.26
	LocationSK	1.62	1.57	1.04	0.30
	breedingStatusBincubating	1.33	1.03	1.28	0.20
	breedingStatusChick-feeding	0.67	0.88	0.76	0.45
	sample_age	-0.003	0.001	-2.91	**0.005**
	LocationNK:breedingStatusBincubating	-3.70	2.00	-1.85	0.07
	LocationSK:breedingStatusBincubating	-2.02	2.28	-0.88	0.38
	LocationNK:breedingStatusChick-feeding	-3.68	1.90	-1.94	0.06
	LocationSK:breedingStatusChick-feeding	-3.56	1.88	-1.90	0.06
lm(AvAgglut~Location+breedingStatus+plasma sample age, data=AggF)<br><br>(Final model: model after elimination of interaction Location:breedingStatus)	(Intercept)	8.45	1.19	7.10	**<0.001**
	LocationNK	-1.07	0.73	-1.46	0.15
	LocationSK	-0.64	0.78	-0.82	0.42
	breedingStatusBincubating	0.34	0.81	0.42	0.67
	breedingStatusChick-feeding	-0.59	0.71	-0.83	0.41
	sample_age	-0.003	0.001	-2.64	**0.01**

Lysis: females only

glm(AvLysis2~Location+breedingStatus+Location:breedingStatus+plasma sample age, binomial, data=FLysis)<br><br>(starting model before elimination of interaction Location:breedingStatus)	(Intercept)	3.93	1.34	2.94	**0.003**
	LocationNK	0.79	1.32	0.60	0.55
	LocationSK	-2.22	1.64	-1.35	0.18
	breedingStatusBincubating	-1.03	0.98	-1.05	0.29
	breedingStatusChick-feeding	-0.50	0.82	-0.62	0.54
	sample_age	-0.005	0.001	-3.82	**<0.001**
	LocationNK:breedingStatusBincubating	-0.56	1.72	-0.33	0.74
	LocationSK:breedingStatusBincubating	5.19	2.20	2.36	**0.02**
	LocationNK:breedingStatusChick-feeding	-1.47	1.66	-0.89	0.37
	LocationSK:breedingStatusChick-feeding	2.99	1.82	1.65	0.10
glm(AvLysis2~Location+breedingStatus+plasma sample	(Intercept)	3.05	1.08	2.81	**0.005**
	LocationNK	-0.15	0.61	-0.24	0.81
	LocationSK	0.91	0.62	1.48	0.14

age, binomial, data=FLysis) (Final model: model after elimination of interaction Location:breedingStatus)	breedingStatusBincubating	-0.28	0.68	-0.42	0.68
	breedingStatusChick-feeding	-0.44	0.63	-0.70	0.49
	sample_age	-0.004	0.001	-3.87	**<0.001**
**Mass: females only**					
lm(mass~Location+ breedingStatus+Location:breedingStatus) (starting model before elimination of interaction Location:breedingStatus)	(Intercept)	23.42	0.30	77.44	< **0.001**
	LocationNK	2.68	0.73	3.69	< **0.001**
	LocationSK	2.96	0.80	3.70	< **0.001**
	breedingStatusBincubating	0.75	0.44	1.69	0.09
	breedingStatusChick-feeding	0.40	0.45	0.88	0.38
	LocationNK:breedingStatusBincubating	-0.89	0.90	-0.99	0.32
	LocationSK:breedingStatusBincubating	-1.31	0.97	-1.35	0.18
	LocationNK:breedingStatusChick-feeding	-1.53	0.90	-1.71	0.09
	LocationSK:breedingStatusChick-feeding	-1.87	0.98	-1.91	0.06
lm(mass~Location+ breedingStatus) (Final model: model after elimination of interaction Location:breedingStatus)	(Intercept)	23.71	0.27	87.69	< **0.001**
	LocationNK	1.76	0.33	5.35	< **0.001**
	LocationSK	1.73	0.35	4.88	< **0.001**
	breedingStatusBincubating	0.45	0.35	1.29	0.20
	breedingStatusChick-feeding	-0.24	0.35	-0.69	0.49

ESM Table 3(b). Coefficient estimates, standard error, t and P values for models with environmental variables (rainfall (mm), average maximum temperature (T_{max}, °C) and average minimum temperature (T_{min}, °C)) as dependent variables and the different sampling time points when we took measurements for two datasets. i) In the case of sexes-combined dataset, we had sampling time points for non-breeding and chick-feeding birds, location, sex and their interaction. ii) In the case of the female-only dataset, we had sampling time points for non-breeding, incubating and chick-feeding birds, location and their interaction. We used these two datasets to test for the potential of these environmental variables as confounding factors in our investigation of the effect of reproduction on immune function that we carried out from January 2011 to March 2014 in three climatically-distinct location: cool and wet South Kinangop (SK), cool and dry North Kinangop (NK) and warm and dry Kedong (KE). Significant P values are in bold

i)

Rainfall: females and males

Model	Explanatory variables	Estimate	SE	t	P
lm(rain~breedingStatus+Location+sex+breedingStatus:Location+breedingStatus:sex+Location:sex) (starting model before elimination of interaction breedingStatus:sex)	(Intercept)	20.05	8.42	2.38	**0.02**
	breedingStatusChick-feeding	21.63	11.75	1.84	0.07
	LocationNK	47.19	17.05	2.77	**0.01**
	LocationSK	67.86	18.69	3.63	**< 0.001**
	sexM	-10.02	11.52	-0.87	0.39
	breedingStatusChick-feeding:LocationNK	-45.73	17.86	-2.56	**0.01**
	breedingStatusChick-feeding:LocationSK	-4.21	19.58	-0.22	0.83
	breedingStatusChick-feeding:sexM	-0.13	14.69	-0.01	0.99
	LocationNK:sexM	5.22	17.73	0.30	0.77
	LocationSK:sexM	-6.14	18.54	-0.33	0.74
lm(rain~breedingStatus+Location+sex+breedingStatus:Location+Location:sex)	(Intercept)	20.08	7.82	2.57	**0.01**
	breedingStatusChick-feeding	21.57	9.59	2.25	**0.03**
	LocationNK	47.21	16.74	2.82	**0.01**

(model after elimination of interaction breedingStatus:sex)	LocationSK	67.89	18.42	3.69	< **0.001**
	sexM	-10.08	9.54	-1.06	0.29
	breedingStatusChick-feeding:LocationNK	-45.75	17.72	-2.58	**0.01**
	breedingStatusChick-feeding:LocationSK	-4.22	19.47	-0.22	0.83
	LocationNK:sexM	5.20	17.48	0.30	0.77
	LocationSK:sexM	-6.18	18.00	-0.34	0.73
lm(rain~breedingStatus+Location+sex+breedingStatus:Location)<br><br>(Final model: model after elimination of interaction Location:sex)	(Intercept)	20.14	7.15	2.82	**0.01**
	breedingStatusChick-feeding	21.57	9.53	2.26	**0.03**
	LocationNK	50.70	12.80	3.96	< **0.001**
	LocationSK	64.46	15.61	4.13	< **0.001**
	sexM	-10.19	7.04	-1.45	0.15
	breedingStatusChick-feeding:LocationNK	-47.11	17.28	-2.73	**0.01**
	breedingStatusChick-feeding:LocationSK	-4.01	19.34	-0.21	0.84
**Average maximum temperature (T_{max} °C): females and males**					
lm(Tmax~breedingStatus+Location+sex+breedingStatus:Location+breedingStatus:sex+Location:sex)<br><br>(starting model before elimination of interaction Location:sex)	(Intercept)	27.14	0.41	66.70	< **0.001**
	breedingStatusChick-feeding	1.86	0.57	3.29	**0.001**
	LocationNK	-3.10	0.82	-3.76	< **0.001**
	LocationSK	-2.76	0.90	-3.06	< **0.001**
	sexM	0.79	0.56	1.42	0.16
	breedingStatusChick-feeding:LocationNK	1.51	0.86	1.76	0.08
	breedingStatusChick-feeding:LocationSK	-1.11	0.95	-1.17	0.24
	breedingStatusChick-feeding:sexM	-0.49	0.71	-0.69	0.49
	LocationNK:sexM	-0.18	0.86	-0.21	0.83
	LocationSK:sexM	-0.57	0.90	-0.64	0.53
lm(Tmax~breedingStatus+Location+sex+breedingStatus:Location+breedingStatus:sex)<br><br>(model after elimination of interaction Location:sex)	(Intercept)	27.19	0.39	69.86	< **0.001**
	breedingStatusChick-feeding	1.91	0.56	3.40	< **0.001**
	LocationNK	-3.20	0.62	-5.13	< **0.001**
	LocationSK	-3.07	0.76	-4.05	< **0.001**
	sexM	0.68	0.50	1.35	0.18
	breedingStatusChick-feeding:LocationNK	1.53	0.84	1.82	0.07
	breedingStatusChick-feeding:LocationSK	-1.08	0.94	-1.15	0.25
	breedingStatusChick-feeding:sexM	-0.60	0.68	-0.87	0.39

lm(Tmax~breedingStatus+Location+sex+breedingStatus:Location)	(Intercept)	27.35	0.35	78.91	< **0.001**
	breedingStatusChick-feeding	1.63	0.46	3.53	< **0.001**
	LocationNK	-3.14	0.62	-5.07	< **0.001**
	LocationSK	-3.04	0.76	-4.02	< **0.001**
(Final model: model after elimination of interaction breedingStatus:sex)	sexM	0.36	0.34	1.05	0.30
	breedingStatusChick-feeding:LocationNK	1.48	0.84	1.77	0.08
	breedingStatusChick-feeding:LocationSK	-1.13	0.94	-1.20	0.23

Average minimum temperature (T_{min} °C): females and males

lm(Tmin~breedingStatus+Location+sex+breedingStatus:Location+breedingStatus:sex+Location:sex)	(Intercept)	10.94	0.34	32.06	< **0.001**
	breedingStatusChick-feeding	-0.37	0.48	-0.77	0.44
	LocationNK	-1.54	0.69	-2.23	0.03
	LocationSK	-5.87	0.76	-7.75	< **0.001**
	sexM	0.66	0.47	1.41	0.16
	breedingStatusChick-feeding:LocationNK	-1.05	0.72	-1.46	0.15
(starting model before elimination of interaction Location:sex)	breedingStatusChick-feeding:LocationSK	1.16	0.79	1.47	0.14
	breedingStatusChick-feeding:sexM	-0.58	0.60	-0.98	0.33
	LocationNK:sexM	-0.87	0.72	-1.21	0.23
	LocationSK:sexM	-0.57	0.75	-0.77	0.45
lm(Tmin~breedingStatus+Location+sex+breedingStatus:Location+breedingStatus:sex)	(Intercept)	11.06	0.33	33.72	< **0.001**
	breedingStatusChick-feeding	-0.31	0.47	-0.65	0.52
	LocationNK	-2.07	0.53	-3.94	< **0.001**
	LocationSK	-6.17	0.64	-9.66	< **0.001**
	sexM	0.41	0.42	0.98	0.33
(model after elimination of interaction Location:sex)	breedingStatusChick-feeding:LocationNK	-0.89	0.71	-1.26	0.21
	breedingStatusChick-feeding:LocationSK	1.20	0.79	1.51	0.13
	breedingStatusChick-feeding:sexM	-0.73	0.58	-1.27	0.21
lm(Tmin~breedingStatus+Location+sex+breedingStatus:Location)	(Intercept)	11.25	0.29	38.41	< **0.001**
	breedingStatusChick-feeding	-0.65	0.39	-1.66	0.10
	LocationNK	-2.00	0.52	-3.82	< **0.001**
	LocationSK	-6.14	0.64	-9.60	< **0.001**
	sexM	0.02	0.29	0.06	0.95

(model after elimination of interaction breedingStatus:sex)	breedingStatusChick-feeding:LocationNK	-0.95	0.71	-1.35	0.18
	breedingStatusChick-feeding:LocationSK	1.14	0.79	1.44	0.15
lm(Tmin~breedingStatus+Location+sex)	(Intercept)	11.24	0.28	40.60	< **0.001**
	breedingStatusChick-feeding	-0.67	0.30	-2.26	**0.03**
Final model: model after elimination of interaction breedingStatus:Location)	LocationNK	-2.56	0.35	-7.23	< **0.001**
	LocationSK	-5.31	0.38	-14.00	< **0.001**
	sexM	0.07	0.29	0.23	0.82

ii)

Rainfall: females only

Model	Explanatory variables	Estimate	SE	t	P
lm(rain~breedingStatus+Location+breedingStatus:Location)	(Intercept)	22.00	10.00	2.20	**0.03**
	breedingStatusBincubating	0.16	14.63	0.01	0.99
	breedingStatusChick-feeding	17.34	14.83	1.17	0.24
	LocationNK	45.46	24.07	1.89	0.06
	LocationSK	53.92	26.45	2.04	**0.04**
(starting model before elimination of interaction breedingStatus:Location)	breedingStatusBincubating:LocationNK	-0.69	29.64	-0.02	0.98
	breedingStatusChick-feeding:LocationNK	-37.86	29.51	-1.28	0.20
	breedingStatusBincubating:LocationSK	-22.91	31.84	-0.72	0.47
	breedingStatusChick-feeding:LocationSK	8.42	31.93	0.26	0.79
lm(rain~breedingStatus+Location)	(Intercept)	25.01	8.98	2.79	**0.01**
	breedingStatusBincubating	-3.23	11.57	-0.28	0.78
	breedingStatusChick-feeding	11.14	11.62	0.96	0.34
(Final model: model after elimination of interaction breedingStatus:Location)	LocationNK	29.70	10.93	2.72	**0.01**
	LocationSK	48.82	11.44	4.27	< **0.001**

Average maximum temperature (T_{max}°C): females only

lm(Tmax~breedingStatus+Location+br	(Intercept)	26.96	0.47	57.54	< **0.001**
	breedingStatusBincubating	5.88	0.69	8.57	< **0.001**

eedingStatus:Location) (starting model and also final model. Interaction breedingStatus:Location is significant)	breedingStatusChick-feeding	2.26	0.70	3.25	**0.00**
	LocationNK	-2.32	1.13	-2.06	**0.04**
	LocationSK	-2.26	1.24	-1.83	0.07
	breedingStatusBincubating:LocationNK	-3.59	1.39	-2.59	**0.01**
	breedingStatusChick-feeding:LocationNK	0.22	1.38	0.16	0.88
	breedingStatusBincubating:LocationSK	-4.08	1.49	-2.74	**0.01**
	breedingStatusChick-feeding:LocationSK	-1.75	1.50	-1.17	0.24

Average minimum temperature (T_{min} °C): females only

lm(Tmin~breedingStatus+Location+breedingStatus:Location) (starting model and also final model. Interaction breedingStatus:Location is significant)	(Intercept)	10.88	0.43	25.34	< **0.001**
	breedingStatusBincubating	-3.01	0.63	-4.80	< **0.001**
	breedingStatusChick-feeding	-0.23	0.64	-0.36	0.72
	LocationNK	-1.14	1.03	-1.10	0.27
	LocationSK	-5.85	1.14	-5.15	< **0.001**
	breedingStatusBincubating:LocationNK	1.71	1.27	1.35	0.18
	breedingStatusChick-feeding:LocationNK	-1.46	1.27	-1.15	0.25
	breedingStatusBincubating:LocationSK	2.89	1.37	2.11	**0.04**
	breedingStatusChick-feeding:LocationSK	0.96	1.37	0.70	0.49
