# Supplementary material for: Immune function differs among tropical environments but is not downregulated during reproduction in three year-round breeding equatorial lark populations
Source: Oecologia. 2021 Oct 12;197(3):599–614. doi: 10.1007/s00442-021-05052-0 (PMC8585810; doi:10.1007/s00442-021-05052-0)
Supplement: Supplementary file 4 — Supplementary file4 (PDF 531 kb) [file 442_2021_5052_MOESM4_ESM.pdf]

Immune function differs among tropical environments but is not downregulated during reproduction in three year-round breeding equatorial lark populations

Submitted to Oecologia

Henry K. Ndithia[1, 2,*], Kevin D. Matson[3], Muchane Muchai[1, 4], B. Irene Tieleman[2]

[1]Ornithology Section, Department of Zoology, National Museums of Kenya, P.O. Box 40658 – 00100 GPO, Nairobi, Kenya

[2]Groningen Institute for Evolutionary Life Sciences, University of Groningen, P.O. Box 11103, 9700 CC Groningen, The Netherlands;

[3]Resource Ecology Group, Department of Environmental Sciences, Wageningen University, Droevendaalsesteegh 3a, 6708 PB Wageningen, The Netherlands

[4]Department of Clinical Studies (Wildlife and Conservation), College of Agriculture and Veterinary Sciences, University of Nairobi. Box 30197-00100, Nairobi, Kenya

*Corresponding author:

Email: hndithia@gmail.com

ESM Table 4. Multiple pairwise comparison of only the significant post hoc results of the interaction of breeding groups (i.e., non-breeding, incubating, chick-feeding) *x* location in female red-capped larks *Calandrella cinerea* for a) average minimum temperature (T_{min}) and b) average maximum temperature (T_{max}), during a study examining variation in immune function between incubating (females), chick-feeding and non-breeding (male and female) red-capped larks in South Kinangop (cool and wet), North Kinangop (cool and dry) and Kedong (warm and dry) in equatorial Kenya. Numbers in the column-row intersection are the P-values and the associated t-values (in bracket) for each paired comparison. Abbreviations in the column headers represent the significantly higher values (at P < 0.05) compared to those in the rows. Legend: CFKE – chick-feeding in Kedong; NBKE – non-breeding in Kedong; NBNK – non-breeding in North Kinangop; INCNK – incubating in North Kinangop; INCKE – incubating in Kedong; CFNK – chick-feeding in North Kinangop; NBSK – non-breeding in South Kinangop; INCSK – incubating in South Kinangop; CFSK – chick-feeding in South Kinangop.

a) T_{min}

	CFKE	NBKE	NBNK	NBKE	INCNK	INCKE	CFKE	CFNK
INCKE	<0.01 (4.24)	<0.01 (4.80)						
NBSK			0.02 (3.34)	<0.01 (5.15)				
INCSK					<0.01 (4.20)	<0.01 (3.90)		
CFSK							<0.01 (6.37)	
CFNK							<0.01 (3.55)	

CFSK				0.09 (2.77)

b) T_{max}

	INCKE	CFKE	INCKE	CFKE
CFKE	<0.01 (5.05)			
NBKE	<0.01 (8.57)	0.02 (3.25)		
INCSK			<0.01 (7.64)	
INCNK			<0.01 (7.30)	
CFSK				<0.01 (4.79)
